# Supplementary material for: Can achievement at medical admission tests predict future performance in postgraduate clinical assessments? A UK-based national cohort study
Source: BMJ Open. 2022 Feb 8;12(2):e056129. doi: 10.1136/bmjopen-2021-056129 (PMC8830227; doi:10.1136/bmjopen-2021-056129)
Supplement: Supplementary data [file bmjopen-2021-056129supp001.pdf]

Supplemental Material for 'Can achievement at medical admissions tests predict future performance in postgraduate clinical assessments? A national cohort study'

Lewis W Paton, I C McManus, Kevin Y F Cheung, Daniel S Smith, Paul A Tiffin

A: Passing each section of the MRCP at the first attempt

Tables A1 – A3 display the results for models predicting passing each section of the Membership of the Royal Colleges of Physicians of the UK (MRCP) at the first attempt. These results are also depicted in Figures 2 and 3.

**Pass at 1st attempt**	**MRCP: Part 1**	
	**Unadjusted ORs**	**ORs adjusted for PEA**
**BMAT**		
**Aptitude and Skills**	1.29 (1.10 to 1.52) p=0.002	1.22 (1.04 to 1.44) p=0.02
**Scientific Knowledge and Applications**	1.58 (1.33 to 1.88) p<0.001	1.49 (1.25 to 1.78) p<0.001
**Writing**	1.02 (0.87 to 1.19) p=0.84	1.00 (0.86 to 1.17) p=0.98
**'Total'**	1.53 (1.30 to 1.80) p<0.001	1.44 (1.22 to 1.69) p<0.001
**UCAT**		
**Abstract Reasoning**	1.08 (0.94 to 1.25) p=0.28	1.04 (0.90 to 1.21) p=0.56
**Decision Analysis**	1.12 (0.95 to 1.32) p=0.17	1.09 (0.92 to 1.28) p=0.32
**Quantitative Reasoning**	1.41 (1.19 to 1.67) p<0.001	1.37 (1.16 to 1.62) p<0.001
**Verbal Reasoning**	1.29 (1.09 to 1.53) p=0.003	1.24 (1.05 to 1.48) p=0.01
**Total**	1.43 (1.18 to 1.73) p<0.001	1.35 (1.11 to 1.63) p=0.003
**'Rebalanced' total**	1.43 (1.19 to 1.72) p<0.001	1.35 (1.12 to 1.63) p<0.01

Table A1. Results from multilevel logistic regression models predicting passing *MRCP: Part 1* at the first attempt, unadjusted and adjusted for prior educational attainment (PEA).

**Pass at 1st attempt**	**MRCP: Part 2**	
	**Unadjusted ORs**	**ORs adjusted for PEA**
**BMAT**		
**Aptitude and Skills**	1.36 (1.06 to 1.75)<br>p=0.02	1.36 (1.05 to 1.75)<br>p=0.02
**Scientific Knowledge and Applications**	1.35 (1.04 to 1.75)<br>p=0.02	1.35 (1.04 to 1.75)<br>p=0.02
**Writing**	1.13 (0.89 to 1.45)<br>p=0.32	1.12 (0.88 to 1.44)<br>p=0.36
**'Total'**	1.44 (1.13 to 1.83)<br>p<0.01	1.44 (1.13 to 1.84)<br>p<0.01
**UCAT**		
**Abstract Reasoning**	0.80 (0.64 to 0.99)<br>p=0.04	0.79 (0.64 to 0.99)<br>p=0.04
**Decision Analysis**	1.02 (0.79 to 1.33)<br>p=0.86	1.02 (0.78 to 1.32)<br>p=0.91
**Quantitative Reasoning**	1.27 (0.98 to 1.65)<br>p=0.07	1.26 (0.97 to 1.65)<br>p=0.08
**Verbal Reasoning**	1.55 (1.18 to 2.04)<br>p=0.002	1.55 (1.17 to 2.04)<br>p<0.01
**Total**	1.17 (0.87 to 1.58)<br>p=0.30	1.15 (0.85 to 1.56)<br>p=0.36
**'Balanced' total**	1.38 (1.03 to 1.86)<br>p=0.03	1.37 (1.02 to 1.85)<br>p=0.04

Table A2. Results from multilevel logistic regression models predicting passing *MRCP: Part 2* at the first attempt, unadjusted and adjusted for prior educational attainment (PEA).

**Pass at 1st attempt**	**MRCP: PACES**	
	**Unadjusted ORs**	**ORs adjusted for PEA**
**BMAT**		
**Aptitude and Skills**	1.35 (1.09 to 1.68)<br>p=0.01	1.34 (1.08 to 1.67)<br>p=0.01
**Scientific Knowledge and Applications**	1.13 (0.90 to 1.42)<br>p=0.30	1.12 (0.89 to 1.41)<br>p=0.34
**Writing**	1.12 (0.88 to 1.43)<br>p=0.34	1.10 (0.87 to 1.41)<br>p=0.42
**'Total'**	1.27 (1.04 to 1.56)<br>p=0.02	1.26 (1.03 to 1.55)<br>p=0.03
**UCAT**		
**Abstract Reasoning**	1.07 (0.88 to 1.32)<br>p=0.49	1.05 (0.86 to 1.29)<br>p=0.63
**Decision Analysis**	1.09 (0.86 to 1.38)<br>p=0.47	1.06 (0.83 to 1.34)<br>p=0.66
**Quantitative Reasoning**	1.17 (0.92 to 1.48)<br>p=0.20	1.15 (0.91 to 1.46)<br>p=0.25
**Verbal Reasoning**	1.38 (1.07 to 1.76)<br>p=0.01	1.34 (1.04 to 1.71)<br>p=0.02
**Total**	1.30 (>1.00 to 1.69)<br>p=0.05	1.25 (0.96 to 1.64)<br>p=0.10
**'Rebalanced' total**	1.39 (1.07 to 1.80)<br>p=0.01	1.34 (1.03 to 1.75)<br>p=0.03

Table A3. Results from multilevel logistic regression models predicting passing *MRCP: PACES* at the first attempt, unadjusted and adjusted for prior educational attainment (PEA).

B: Imputed results

Tables B1-B3 display results from multilevel logistic models fitted to both non-imputed and imputed data. These models are adjusted for prior educational attainment.

**Pass at 1st attempt**	**MRCP: Part 1**	
	**Adjusted ORs**	
	**Non-imputed**	**Imputed**
**BMAT**		
**Aptitude and skills**	1.22 (1.04 to 1.44) p=0.02	1.25 (1.08 to 1.45) p<0.01
**Scientific knowledge and applications**	1.49 (1.25 to 1.78) p<0.001	1.52 (1.30 to 1.78) p<0.001
**Writing**	1.00 (0.86 to 1.17) p=0.98	1.08 (0.94 to 1.24) p=0.30
**'Total'**	1.44 (1.22 to 1.69) p<0.001	1.48 (1.28 to 1.71) p<0.001
**UCAT**		
**Abstract Reasoning**	1.04 (0.90 to 1.21) p=0.56	1.07 (0.98 to 1.17) p=0.14
**Decision Analysis**	1.09 (0.92 to 1.28) p=0.32	1.10 (<1.00 to 1.21) p=0.06
**Quantitative Reasoning**	1.37 (1.16 to 1.62) p<0.001	1.31 (1.18 to 1.45) p<0.001
**Verbal Reasoning**	1.24 (1.05 to 1.48) p=0.01	1.30 (1.17 to 1.43) p<0.001
**Total**	1.35 (1.11 to 1.63) p=0.003	1.37 (1.22 to 1.54) p<0.001
**'Rebalanced' total**	1.35 (1.12 to 1.63) p<0.01	1.40 (1.25 to 1.56) p<0.001

Table B1. Comparison of odds ratios on non-imputed and imputed data, predicting passing *MRCP: Part 1* at the first attempt. All models are adjusted for prior educational attainment.

Pass at 1st attempt	MRCP: Part 2	
	Adjusted ORs	
	Non-imputed	Imputed
**BMAT**		
**Aptitude and skills**	1.36 (1.05 to 1.75) p=0.02	1.35 (1.09 to 1.67) p=0.01
**Scientific knowledge and applications**	1.35 (1.04 to 1.75) p=0.02	1.33 (1.06 to 1.65) p=0.01
**Writing**	1.12 (0.88 to 1.44) p=0.36	1.14 (0.92 to 1.40) p=0.24
**'Total'**	1.44 (1.13 to 1.84) p<0.01	1.40 (1.13 to 1.72) p<0.01
**UCAT**		
**Abstract Reasoning**	0.79 (0.64 to 0.99) p=0.04	1.00 (0.87 to 1.16) p=0.97
**Decision Analysis**	1.02 (0.78 to 1.32) p=0.91	0.98 (0.83 to 1.12) p=0.66
**Quantitative Reasoning**	1.26 (0.97 to 1.65) p=0.08	1.22 (1.04 to 1.44) p=0.02
**Verbal Reasoning**	1.55 (1.17 to 2.04) p=0.002	1.47 (1.25 to 1.74) p<0.001
**Total**	1.15 (0.85 to 1.56) p=0.36	1.26 (1.05 to 1.52) p=0.01
**'Rebalanced' total**	1.37 (1.02 to 1.85) p=0.04	1.42 (1.19 to 1.70) p<0.001

Table B2. Comparison of odds ratios on non-imputed and imputed data, predicting passing *MRCP: Part 2* at the first attempt. All models are adjusted for prior educational attainment.

**Pass at 1st attempt**	**MRCP: PACES**	
	**Adjusted ORs**	
	**Non-imputed**	**Imputed**
**BMAT**		
**Aptitude and skills**	1.34 (1.08 to 1.67) p=0.01	1.20 (1.03 to 1.40) p=0.02
**Scientific knowledge and applications**	1.12 (0.89 to 1.41) p=0.34	1.12 (0.94 to 1.35) p=0.21
**Writing**	1.10 (0.87 to 1.41) p=0.42	1.00 (0.84 to 1.19) p=0.99
**'Total'**	1.26 (1.03 to 1.55) p=0.03	1.19 (1.03 to 1.37) p=0.02
**UCAT**		
**Abstract Reasoning**	1.05 (0.86 to 1.29) p=0.63	1.10 (0.97 to 1.24) p=0.13
**Decision Analysis**	1.06 (0.83 to 1.34) p=0.66	1.14 (>1.00 to 1.30) p=0.05
**Quantitative Reasoning**	1.15 (0.91 to 1.46) p=0.25	1.07 (0.94 to 1.23) p=0.31
**Verbal Reasoning**	1.34 (1.04 to 1.71) p=0.02	1.15 (>1.00 to 1.32) p=0.05
**Total**	1.25 (0.96 to 1.64) p=0.10	1.22 (1.05 to 1.42) p=0.01
**'Rebalanced' total**	1.34 (1.03 to 1.75) p=0.03	1.22 (1.05 to 1.41) p=0.01

Table B3. Comparison of odds ratios on non-imputed and imputed data, predicting passing *MRCP: PACES* at the first attempt. All models are adjusted for prior educational attainment.

C: Results from 'score relative to pass'

Tables C1 – C3 display results from linear regression models predicting score relative to pass at each section of the MRCP(UK) at the first attempt. Regression coefficients are shown unadjusted and adjusted for prior educational attainment.

Score relative to pass	MRCP: Part 1	
	Unadjusted β	β adjusted for PEA
**BMAT**		
Aptitude and Skills	1.25 (0.68 to 1.82) p<0.001	1.07 (0.49 to 1.65) p<0.001
Scientific Knowledge and Applications	2.02 (1.43 to 2.60) p<0.001	1.83 (1.24 to 2.42) p<0.001
Writing	-0.04 (-0.60 to 0.51) p=0.88	-0.06 (-0.62 to 0.49) p=0.83
'Total'	1.95 (1.40 to 2.50) p<0.001	1.76 (1.20 to 2.32) p<0.001
**UCAT**		
Abstract Reasoning	0.25 (-0.25 to 0.75) p=0.38	0.14 (-0.36 to 0.64) p=0.59
Decision Analysis	0.46 (-0.10 to 1.03) p=0.11	0.37 (-0.19 to 0.94) p=0.19
Quantitative Reasoning	1.62 (1.03 to 2.21) p<0.001	1.51 (0.92 to 2.10) p<0.001
Verbal Reasoning	1.03 (0.43 to 1.62) p<0.001	0.91 (0.31 to 1.50) p<0.01
Total	1.46 (0.80 to 2.11) p<0.001	1.27 (0.61 to 1.93) p<0.001
'Rebalanced' total	1.47 (0.82 to 2.12) p<0.001	1.30 (0.64 to 1.95) p<0.001

Table C1. Results from multilevel linear regression models predicting score relative to pass on *MRCP: Part 1* at the first attempt, unadjusted and adjusted for prior educational attainment (PEA).

**Score relative to pass**	**MRCP: Part 2**	
	**Unadjusted β**	**β adjusted for PEA**
**BMAT**		
**Aptitude and Skills**	1.80 (1.27 to 2.32) p<0.001	1.75 (1.21 to 2.28) p<0.001
**Scientific Knowledge and Applications**	1.71 (1.16 to 2.26) p<0.001	1.71 (1.16 to 2.27) p<0.001
**Writing**	0.30 (-0.26 to 0.87) p=0.29	0.38 (-0.19 to 0.95) p=0.19
**'Total'**	1.90 (1.42 to 2.38) p<0.001	1.87 (1.37 to 2.36) p<0.001
**UCAT**		
**Abstract Reasoning**	0.05 (-0.45 to 0.55) p=0.84	0.04 (-0.47 to 0.55) p=0.87
**Decision Analysis**	0.37 (-0.21 to 0.95) p=0.21	0.38 (-0.20 to 0.97) p=0.20
**Quantitative Reasoning**	1.14 (0.53 to 1.74) p<0.001	1.17 (0.56 to 1.78) p<0.001
**Verbal Reasoning**	1.66 (1.04 to 2.27) p<0.001	1.62 (1.00 to 2.25) p<0.001
**Total**	1.22 (0.56 to 1.88) p<0.001	1.25 (0.58 to 1.92) p<0.001
**'Rebalanced' total**	1.60 (0.95 to 2.26) p<0.001	1.69 (1.03 to 2.36) p<0.001

Table C2. Results from multilevel linear regression models predicting score relative to pass on *MRCP: Part 2* at the first attempt, unadjusted and adjusted for prior educational attainment (PEA).

**Score relative to pass**	**MRCP: PACES**	
	**Unadjusted β**	**β adjusted for PEA**
**BMAT**		
**Aptitude and Skills**	2.37 (0.88 to 2.89) p<0.01	2.36 (0.85 to 3.87) p<0.01
**Scientific Knowledge and Applications**	1.68 (0.11 to 3.36) p=0.04	1.66 (0.07 to 3.26) p=0.04
**Writing**	1.22 (-0.47 to 2.92) p=0.16	1.17 (-0.54 to 2.88) p=0.18
**'Total'**	2.19 (0.80 to 3.57) p<0.01	2.19 (0.78 to 3.60) p<0.01
**UCAT**		
**Abstract Reasoning**	0.68 (-0.77 to 2.14) p=0.36	0.62 (-0.85 to 2.10) p=0.41
**Decision Analysis**	0.86 (-0.84 to 2.55) p=0.32	0.77 (-0.96 to 2.50) p=0.38
**Quantitative Reasoning**	0.79 (-0.91 to 2.49) p=0.36	0.74 (-0.97 to 2.44) p=0.40
**Verbal Reasoning**	3.00 (1.33 to 4.67) p<0.001	2.99 (1.28 to 4.70) p<0.001
**Total**	2.18 (0.36 to 4.00) p=0.02	2.14 (0.26 to 4.02) p=0.03
**'Rebalanced' total**	2.88 (1.09 to 4.66) p<0.01	2.89 (1.04 to 4.74) p<0.01

Table C3. Results from multilevel linear regression models predicting score relative to pass on *MRCP: PACES* at the first attempt, unadjusted and adjusted for prior educational attainment (PEA).
