# Supplementary material for: Can achievement at medical admission tests predict future performance in postgraduate clinical assessments? A UK-based national cohort study
Source: BMJ Open. 2022 Feb 8;12(2):e056129. doi: 10.1136/bmjopen-2021-056129 (PMC8830227; doi:10.1136/bmjopen-2021-056129)
Supplement: Author's manuscript [file bmjopen-2021-056129.draft_revisions.pdf]

BMJ Open

BMJ Open is committed to open peer review. As part of this commitment we make the peer review history of every article we publish publicly available.

When an article is published we post the peer reviewers' comments and the authors' responses online. We also post the versions of the paper that were used during peer review. These are the versions that the peer review comments apply to.

The versions of the paper that follow are the versions that were submitted during the peer review process. They are not the versions of record or the final published versions. They should not be cited or distributed as the published version of this manuscript.

BMJ Open is an open access journal and the full, final, typeset and author-corrected version of record of the manuscript is available on our site with no access controls, subscription charges or pay-per-view fees (http://bmjopen.bmj.com).

If you have any questions on BMJ Open's open peer review process please email info.bmjopen@bmj.com

BMJ Open

Can achievement at medical admissions tests predict future performance in postgraduate clinical assessments? A UK-based national cohort study

Journal:	*BMJ Open*
Manuscript ID	bmjopen-2021-056129
Article Type:	Original research
Date Submitted by the Author:	04-Aug-2021

Complete List of Authors:	Paton, Lewis; University of York, Health Sciences<br>McManus, Ian; University College London, Psychology<br>Cheung, Kevin; University of Cambridge, Cambridge Assessment<br>Smith, Daniel; General Medical Council, Education and Standards<br>Tiffin, Paul; University of York, Health Sciences

Keywords:	MEDICAL EDUCATION & TRAINING, GENERAL MEDICINE (see Internal Medicine), EDUCATION & TRAINING (see Medical Education & Training)

SCHOLARONE™
Manuscripts

I, the Submitting Author has the right to grant and does grant on behalf of all authors of the Work (as defined in the below author licence), an exclusive licence and/or a non-exclusive licence for contributions from authors who are: i) UK Crown employees; ii) where BMJ has agreed a CC-BY licence shall apply, and/or iii) in accordance with the terms applicable for US Federal Government officers or employees acting as part of their official duties; on a worldwide, perpetual, irrevocable, royalty-free basis to BMJ Publishing Group Ltd ("BMJ") its licensees and where the relevant Journal is co-owned by BMJ to the co-owners of the Journal, to publish the Work in this journal and any other BMJ products and to exploit all rights, as set out in our licence.

The Submitting Author accepts and understands that any supply made under these terms is made by BMJ to the Submitting Author unless you are acting as an employee on behalf of your employer or a postgraduate student of an affiliated institution which is paying any applicable article publishing charge ("APC") for Open Access articles. Where the Submitting Author wishes to make the Work available on an Open Access basis (and intends to pay the relevant APC), the terms of reuse of such Open Access shall be governed by a Creative Commons licence – details of these licences and which Creative Commons licence will apply to this Work are set out in our licence referred to above.

Other than as permitted in any relevant BMJ Author's Self Archiving Policies, I confirm this Work has not been accepted for publication elsewhere, is not being considered for publication elsewhere and does not duplicate material already published. I confirm all authors consent to publication of this Work and authorise the granting of this licence.

For peer review only

Can achievement at medical admissions tests predict future performance in postgraduate clinical assessments? A UK-based national cohort study

Lewis W Paton, I C McManus, Kevin Y F Cheung, Daniel T Smith, Paul A Tiffin

LWP (corresponding author): Department of Health Sciences, University of York, UK, ORCID: https://orcid.org/0000-0002-3328-5634, lewis.paton@york.ac.uk

ICM: Research Department of Medical Education, University College London, UK. ORCID: https://orcid.org/0000-0003-3510-4814

KYFC: Cambridge Assessment English, University of Cambridge, UK. ORCID: https://orcid.org/0000-0002-9548-2932

DTS: General Medical Council, UK. ORCID: https://orcid.org/0000-0003-1215-5811

PAT: Department of Health Sciences, University of York and Health Professions Education Unit, Hull York Medical School, UK. ORCID: https://orcid.org/0000-0003-1770-5034

For peer review only - http://bmjopen.bmj.com/site/about/guidelines.xhtml

ABSTRACT

Objective To determine if scores on two undergraduate admissions tests (BioMedical Admissions Test, BMAT and University Clinical Aptitude Test, UCAT) predict performance on the postgraduate Membership of the Royal College of Physicians (MRCP) examination, including the clinical examination Practical Assessment of Clinical Examination Skills (PACES).

Design National cohort study.

Setting Doctors who graduated medical school between 2006 and 2018.

Participants 3,045 doctors who had sat BMAT, UCAT and the MRCP.

Primary outcome measures Passing each section of the MRCP at the first attempt, including the clinical assessment PACES.

Results Several BMAT and UCAT subtest scores displayed incremental predictive validity for performance on the first two (written) parts of the MRCP. Only *aptitude and skills* on BMAT (OR 1.34, 1.08-1.67, p=0.01) and *verbal reasoning* on UCAT (OR 1.34, 1.04-1.71, p=0.02) incrementally predicted passing PACES at the first attempt.

Conclusions Only subtests assessing 'thinking skills' or 'verbal reasoning' predicted performance in the clinical assessment, PACES. This implies these abilities may be the most important cognitive attributes, of those routinely assessed at selection, for predicting future clinical performance. Selectors may wish to consider placing particular weight on scales assessing these attributes if they wish to select applicants likely to become more competent clinicians. These results are potentially relevant in an international context too, since many admissions tests used globally, such as the Medical College Admission Test, assess similar abilities.

For peer review only - http://bmjopen.bmj.com/site/about/guidelines.xhtml

Strengths and limitations of this study

- We had access to a national sample of medical graduates.
- Restricting our analyses to those who had sat both BMAT and UCAT allowed for a comparison of results across the two admissions tests.
- While our results are likely to be generalisable to all applicants who sit BMAT, it is unclear if this is the case for all applicants who sit UCAT.
- While a substantial proportion of doctors sit the MRCP as part of their training, the generalisability of our results to other Royal College examinations is unclear.

For peer review only

For peer review only - http://bmjopen.bmj.com/site/about/guidelines.xhtml

Traditionally, access to medical school has been based on educational attainment in secondary (high) school. Academic entry standards have partly been driven by strong competition for places, as well as the intellectual demands of the courses. Moreover, prior educational attainment has been shown to predict performance in undergraduate and postgraduate medical training [1-6]. Applicants to medical school are also required to sit additional standardised tests as part of the selection process. These have been introduced to help selectors further differentiate between educationally high performing candidates and choose those considered to have the aptitude for a medical career. At times it has also been hoped that such tests will facilitate widening access to medicine, if they are less sensitive to certain sociodemographic characteristics, compared to traditional metrics of attainment [7]. As with educational attainment, scores from assessments such as BioMedical Admissions Test (BMAT) [8], Hamburg Medical School Natural Science Test [9], Health Professions Admission test-Ireland [10], Medical College Admissions Test (MCAT) [11], Undergraduate Medicine and Health Sciences Admission Test [12] and Universities Clinical Admissions Test (UCAT- previously known as the United Kingdom Clinical Aptitude Test) [13] have all been shown to have some ability to predict academic achievement in medical school.

The two main selection assessments currently used for entry into undergraduate medicine in UK medical schools are BMAT [14] and UCAT [15]. These are used in conjunction with other selection measures, such as interviews (including multiple mini-interviews), personal statements, references and educational achievement. For UK medical schools, the Medical Schools Council maintains data on the admissions requirements for each cycle and publishes it on their website for prospective applicants [16]. Applicants to undergraduate medicine in the UK may apply to up to four different universities in a particular application cycle. As such, candidates may sit one or both of BMAT and UCAT, depending on the entry requirements of their chosen medical schools. These entry requirements may change year on year. Successful

For peer review only - http://bmjopen.bmj.com/site/about/guidelines.xhtml

applicants to medical school then embark on an undergraduate medical course generally consisting of five years. Medical graduates then undertake the two-year Foundation Programme, before embarking on specialty training in their chosen field of medicine. Each specialty is aligned with a particular Royal College, and as part of their specialty training, doctors must pass the Royal College membership examinations. Some of these exams can be taken during the Foundation Programme prior to commencing specialty training.

As of the 2020 admissions cycle, BMAT is used by nine UK medical schools as part of their selection process [14]. It is a two-hour test which currently consists of three sections: *thinking skills, scientific knowledge and applications* and *writing task.* Prior to 2020, the first section was known as *aptitude and skills* and tested ‘problem solving and understanding argument’. S*cientific knowledge and applications* tests ‘the ability to apply scientific knowledge typically covered in school science and mathematics by the age of 16’. The first two sections of BMAT are multiple-choice questionnaires, and in the third section (*writing*), examinees must answer one extended question. For applicants to UK medical schools, in each application cycle there are two sessions in which candidates can sit BMAT, but applicants can only take BMAT once per application cycle. Raw scores from *aptitude and skills* and *scientific knowledge and applications* are converted to a 9-point Rasch calibrated ratio scale to one decimal place. Prior to 2010, *writing* scores were reported on a 15-point ordinal scale. Since 2010, examinees have received two scores for *writing*: a score for content on a 10-point ordinal scale (*writing: content)*, and a score for quality of written English, on a 5-point ordinal scale *writing: English)*. Previous research has shown that *aptitude and skills* could be conceptualised as measuring ‘thinking skills’ [17].

As of the 2020 admissions cycle, sitting the UCAT is currently required for application by 30 UK medical schools [15]. Candidates can sit the test once per application cycle, and their scores are valid for one year. It is a two-hour, computer-based test, and presently consists of five

For peer review only - http://bmjopen.bmj.com/site/about/guidelines.xhtml

sections: *abstract reasoning*, *decision making, quantitative reasoning*, *verbal reasoning* and *a situational judgement test (SJT). Abstract reasoning* 'assesses the use of convergent and divergent thinking to infer relationships from information', *decision making* 'assesses the ability to make sound decisions and judgements using complex information', *quantitative reasoning* 'assesses the ability to critically evaluate information presented in numerical form' and *verbal reasoning* 'assesses the ability to critically evaluate information presented in written form' [18]. Prior to 2017 *decision analysis* was assessed rather than *decision making*. Each subtest of the UCAT (not including the SJT) is presented in multiple-choice format, and is scored on a scale from 300 to 900. The 'UCAT total score', which is most commonly used by medical school selectors to aid decisions, thus ranges from 1200 to 3600. The SJT was introduced in 2014, having been piloted in 2013, 'measures the capacity to understand real world situations and to identify critical factors and appropriate behaviour in dealing with them'.

It has previously been reported that scores on BMAT are predictive of first year examination scores [8], and that UCAT scores are predictive of undergraduate performance throughout undergraduate medical school [13 19 20]. In the case of the UCAT, the ability of the scores have been shown to demonstrate incremental validity, above and beyond that provided by prior educational achievement [13]. Additionally, there are some indications that the admissions test scores are associated with performance in the written components of two UK Royal College membership examinations (equivalent to US board examination) [21 22]. Thus, there is some evidence that these assessments add value to the selection process when determining which applicants may be best suited to the academic challenges of early medical training. However, performance in written examinations is perhaps less important than how an individual behaves in actual clinical practise. Whilst relevant semantic knowledge is vital in order to deliver safe and effective care, other skills, such as problem-solving ability, are also likely to

For peer review only - http://bmjopen.bmj.com/site/about/guidelines.xhtml

be crucial. Potential doctors are increasingly assessed on these abilities in a variety of ways, notably by selection assessments. Indeed, the admissions tests currently most widely used consist of a number of subsections, some of which aim to evaluate more fluid concepts of cognitive ability, in addition to sections evaluating the ability to recall factual knowledge.

However, linking performance on admissions assessments to aspects of actual clinical care and patient outcomes is extremely challenging for several reasons. One major barrier would be distinguishing between the impact of the individual doctor, versus the team or service, on a care outcome. Nevertheless, simulation-based examinations may serve as a plausible proxy for actual clinical behaviour, albeit reflecting 'maximal' rather than 'typical' performance. Such examinations are commonly taken as part of postgraduate medical training. One of the most widely taken Royal College examinations in the UK is the *Membership of the Royal Colleges of Physicians of the UK (MRCP(UK))* [23]. The MRCP(UK), referred to hereafter as the MRCP, consists of three parts: *Part 1*, *Part 2*, and the *Practical Assessment of Clinical Examination Skills (PACES).* Trainees must complete all parts of the examination to advance to higher specialty training in the UK. *Part 1,* which consists of best-of-five, single-best answer questions with a short clinical scenario, is designed to assess 'a broad range of appropriate knowledge'. *Part 2* is designed to test the acquisition of 'a representative sample of medical knowledge, skills and behaviour', and while also best-of-five, single-best-answer in format, has much more extended clinical scenarios often containing biochemical and haematological data, as well as clinical images. Part 2 involves a higher level of clinical judgement, often reconciling conflicting or irrelevant information. Both part 1 and part 2 questions are in multiple-choice format . Candidates in part 1 and part 2 are given an equated 'overall scaled score', and the 'vast majority' of candidates score between 200 and 800 on each section. The current pass mark for Part 1 is 540, and for Part 2 is 454 [24]. There is some evidence that UCAT scores correlate with scores on Part 1 of the MRCP [21]. The clinical

For peer review only - http://bmjopen.bmj.com/site/about/guidelines.xhtml

assessment *PACES* is designed to test 'clinical knowledge and skills', and involves candidates examining real patients with real clinical signs in various organ systems, as well as taking histories and carrying out clinical communication, usually with surrogate patients. Participants must pass each of the seven skills assessed, as well as achieving an overall score, currently set at 130, in order to pass *PACES* [24]. Candidates must pass *Part 1* before attempting *Part 2* or *PACES*. The latter two sections of the MRCP can be taken in either order. Currently, no evidence relates to how BMAT or UCAT scores relate to performance on PACES.

The creation of the UK Medical Education Database (UKMED) [25], has generated a longitudinal linked dataset relating to individuals from application to medical school, through graduation and into post qualification medical training. The UKMED has now matured to the point that there is a valuable opportunity to evaluate the extent to which performance at selection into medical school is reflected in performance in clinical assessments, many years later, following graduation. Specifically, it would be possible to evaluate which components of selection assessments have the most incremental predictive validity in this regard. This study thus had the following specific aims:

- To assess to what extent performance on the different components of BMAT and UCAT predict performance on the MRCP examinations, with a particular focus on the clinical assessment PACES.
- To determine if this predictive validity was incremental, over that provided by secondary (high) school educational achievement- the other main intellectual ability measure commonly used in the selection of medical students.

The findings would have clear implications for how scores from such tools are used within the selection process, and in particular, the relative weight that should be placed on those

For peer review only - http://bmjopen.bmj.com/site/about/guidelines.xhtml

components mainly testing semantic recall, compared to problem-solving skills. In the context of UK medical selection, understanding the predictive profiles of BMAT and UCAT would help selectors choose an admission test they felt best suited their local requirements. However, as similar cognitive assessments are used globally in medical selection our results would have international relevance. For example, the MCAT, used widely across the United States and Canada, is broadly similar to the BMAT, in that it consists of sections assessing problem solving skills and other sections which assess recall of scientific knowledge. The predictive validity of the MCAT has been widely reported for medical school outcomes and United States Medical Licensing Examination (USMLE) outcomes.[26 27] While direct comparisons of training pathways is not possible, the outcomes considered in this study, performance on the Royal College examination MRCP, are more broadly aligned with certification examination performance in the United States. Determining whether scores from admissions tests, and if so which components of the test, can predict such distal outcomes would have international implications too.

METHODS

Note that all data and results presented in this paper are blunted in line with UKMED statistical disclosure controls [28].

Data availability and preparation

All data used in the analyses were obtained from the UK Medical Education Database (UKMED) [25]. Data were available for 69, 885 medical graduates between 2006 and 2018. Of these, 13,090 graduates had MRCP outcome data. We restricted analyses to those students who had sat both BMAT and UCAT; that is 3,045 graduates. This was to enable a comparison of the predictive validity between the two admissions tests. Figure 1 shows the

For peer review only - http://bmjopen.bmj.com/site/about/guidelines.xhtml

flow of data through this study. It should be noted that the majority of graduates who had sat BMAT had also sat the UCAT, though the reverse was not true.

[INSERT FIGURE 1 HERE]

Outcome variables

The primary outcomes of interest were the odds of passing each section of the MRCP at the first attempt. Performance at the first attempt at each section of the MRCP was chosen as this has been shown to be a good indicator of overall performance [29]. Predictions relating to a secondary outcome, the continuous 'score relative to pass at first sitting', for the exam components were also analysed. The results relating to these latter, continuous outcomes are presented in the Supplemental Material.

Predictor variables

In order to make our results relevant to selectors, performance at the most recent attempt at each section of both admissions tests was used, since it is these scores that are associated with admission to medical school. The subtest scores of BMAT (*aptitude and skills*, *scientific knowledge and applications* and *writing*) and the UCAT (*abstract reasoning*, *decision analysis*, *quantitative reasoning* and *verbal reasoning*) were standardised as z-scores (mean 0, standard deviation 1) within each cohort of test takers (including unsuccessful applicants) and used as independent variables in our model. This standardisation process was intended to increase the comparability of selection assessment scores over time, as these are known to change.

For peer review only - http://bmjopen.bmj.com/site/about/guidelines.xhtml

As medical school selectors often base decisions on combined scores on the admissions tests, we also included a number of summed scales. We included summed BMAT *aptitude and skills and scientific knowledge and applications* score, denoted 'total BMAT score'. We also included *total UCAT score* on all four scales. Additionally, it has been previously been shown that UCAT can be conceptualised as measuring two dimensions of cognitive functioning; verbal and non-verbal reasoning [30]. Thus, we also created a 'rebalanced' total UCAT score in order to adjust for the high relative weighting on non-verbal reasoning created by simply summing the scores from all four subtests. This 'rebalanced' score is the average of the three non-verbal scales (*abstract reasoning, decision analysis, quantitative reasoning*) added to the *verbal reasoning* score. All these summary scores were standardised within each cohort.

It was important to assess the *incremental* predictive validity of the two admissions tests, over and above that provided by conventional education achievement at secondary school. Therefore, two measures of prior academic attainment were used as covariates in the predictive models evaluated. Firstly, A-level performance was used for those students from English secondary schools. As in previous research [19], we used each entrant's best three A-level results, excluding 'General Studies'. We restricted analyses to those with A-levels sat prior to the introduction of A* grades in 2010. Achieved grades were awarded a tariff score (A=10, B=8 etc.). Thus, the maximum tariff for each entrant was 30. The tariff scores were then standardised as z-scores by cohort of applicants. The second measure of prior academic attainment we used was General Certificate of Secondary Education (GCSE) performance. Data were available on the sum of the nine best GCSE grades each candidate scored, where A*=6, A=5, B=4, and so on. 'Double awards' were counted as two separate GCSEs. Summed performance was standardised as z-scores by cohort of applicants. The dates of sitting of GCSEs were not available in the data. Thus, we determined cohorts by year of first sitting of an admissions test, assuming that the majority of candidates from the same GCSE cohort will

For peer review only - http://bmjopen.bmj.com/site/about/guidelines.xhtml

belong to the same admissions test cohort. This approach would not achieve perfect accuracy, since some individuals will choose to take a gap year or defer entry into university. However, it represented the optimum way, pragmatically to estimate the year of GCSE sitting, in order to attempt to adjust for 'grade inflation'.

Data analysis

Multilevel logistic regression models were built for the dichotomous outcome 'pass at first sitting', for each section of the MRCP. Multilevel models were used to allow the effects of the predictors to vary across medical school attended (via a random intercept), as previous research has shown that average MRCP scores are, to some extent, related to the particular medical school attended [31].

Models were built for each subtest score of BMAT and the UCAT, as well as the summary measures of the overall scores described above. Models were built that were both unadjusted and adjusted for prior educational attainment (GCSE and A-level performance). Only information from graduates with complete prior educational data were included in the models, to ensure full nesting of the models. Therefore, n=1,320 individuals were included in our final analysis, including 1,315 who had data for Part 1 of the MRCP, 825 who had Part 2 data, and 460 who had PACES data. Of these, at the first attempt, 975 passed Part 1, 715 passed Part 2, and 310 passed PACES.

Missing data

Multiple imputation was used as a form of sensitivity analysis to assess the impact of missing prior educational data (GCSEs and A-levels) on our results. Standardised GCSE and A-level scores were imputed using multiple imputations by chained equations with linear regression from other educational data and sociodemographic variables. The number of imputations was chosen to ensure stability of results. We analysed the imputed dataset in the manner described

For peer review only - http://bmjopen.bmj.com/site/about/guidelines.xhtml

above, and compared regression coefficients with the models performed on non-imputed data. This allowed us to assess the impact of missing educational data on our results.

All analyses were performed in the Health Informatics Centre Safe Haven using Stata version 14 [32].

Patient and Public Involvement

There was no patient or public involvement in this study.

RESULTS

Descriptive statistics

[INSERT TABLE 1 HERE]

Table 1 displays descriptive statistics for our cohort. Compared to the wider cohort of medical graduates, those who sat both BMAT and UCAT had higher academic attainment. These differences were all statistically significant at the p=0.05 level on Mann-Whitney U testing. Of those graduates who had sat both BMAT and UCAT, those who had also sat the MRCP had somewhat higher scores on the first two sections of BMAT, but lower scores on the writing component of BMAT as well as each section of the UCAT. Again, these differences were statistically significant at the p=0.05 level on formal testing.

Predictive validity of BMAT and UCAT for MRCP performance

Figure 2 displays the predictive and incremental validity for BMAT subtest and summary scores for passing each section of the MRCP at the first attempt. Figure 3 displays the same values for the UCAT subtest and summary scores. In both figures, the circle represents the

For peer review only - http://bmjopen.bmj.com/site/about/guidelines.xhtml

odds ratio from the univariable (or unadjusted) multilevel logistic regression models. The triangle represents the odds ratio from the multivariable (or adjusted) models. Tables displaying these results are available in the Supplemental Material.

[INSERT FIGURE 2 HERE]

As can be seen in Figure 2, passing the first part of the MRCP is incrementally predicted by scores on *aptitude and skills* (OR 1.22, 1.04 to 1.44, p=0.02) and scores on *scientific knowledge and applications* (OR 1.49, 1.25 to 1.78, p<0.001). This later result can be interpreted as follows. For every standard deviation above the mean an individual scored on *scientific knowledge and applications,* their odds of passing Part 1 of the MRCP at the first attempt increased by around 49%. Similarly, performance on both *aptitude and skills* (OR 1.36, 1.05 to 1.75, p=0.02*)* and *scientific knowledge and applications* (OR 1.35, 1.04 to 1.75, p=0.02*)* incrementally predicts passing Part 2 of the MRCP at the first attempt. When it comes to passing the clinical assessment PACES, evidence of predictive validity was only observed for only *aptitude and skills* (OR 1.34, 1.08 to 1.67, p=0.01), and this result is independent of prior educational attainment. 'Total' BMAT score (that is, total score across the first two sections) is a significant predictor of passing all sections of the MRCP at the first attempt. In contrast, the writing component of BMAT is non-significant for all the outcome variables analysed here.

[INSERT FIGURE 3 HERE]

For peer review only - http://bmjopen.bmj.com/site/about/guidelines.xhtml

In the case of UCAT (Figure 3), *quantitative reasoning* (OR 1.37, 1.16 to 1.62, p<0.001) and *verbal reasoning* (OR 1.24, 1.05 to 1.48, p=0.01) scores incrementally predicted passing the first, knowledge-based, part of the MRCP at the first attempt. Higher v*erbal reasoning* scores (OR 1.55, 1.17 to 2.04, p<0.01) are associated with higher odds of passing Part 2 (applied knowledge) component of the MRCP at the first attempt. However, a higher score on the UCAT *abstract reasoning* subtest was associated with *lower* odds of passing MRCP Part 2 at the first attempt, independently of PEA (OR 0.79, 0.64 to 0.99, p=0.04). Only the UCAT *verbal reasoning* scores were statistically significantly associated with the odds of passing the clinical assessment, PACES, at first attempt (OR 1.34, 1.04 to 1.71, p=0.02). In this case, the odds of passing the PACES at first attempt increased by around a third for every standard deviation above the mean achieved on the UCAT *verbal reasoning* subtest, for applicants sitting the test. The total UCAT score is a significant independent predictor of passing part 1 of the MRCP, but not subsequent sections of the exam. However, the 'rebalanced' UCAT score, which weights verbal and non-verbal performance equally, is a statistically significant independent predictor of passing all sections of the MRCP, including PACES (OR 1.34, 1.03 to 1.75, p=0.03). Indeed, the odds of passing the PACES at first attempt increase by around a third for every standard deviation above the mean achieved for applicants sitting the test. No statistically significant associations with the outcomes of interest and the UCAT *decision analysis* scores were observed.

Imputation of prior educational achievement

In those who sat both BMAT and UCAT, missing values of A-levels and GCSE scores were imputed. We used chained imputation methods, building models on available sociodemographic variables and educational variables, where present. Imputation results stabilised after 20 iterations. Logistic regression models, controlling for the influence of A-levels and GCSEs, were fitted to the imputed data set to assess the incremental predictive

For peer review only - http://bmjopen.bmj.com/site/about/guidelines.xhtml

validity of each scale score for predicting whether an individual passed each section of the MRCP on first attempt. As a form of sensitivity analysis, regression coefficients were compared to coefficients obtained when fitting the same model to the non-imputed data (as presented in Figures 2 and 3). Full results from models on imputed data are available in the Supplemental Material.

In general, the results from models fitted to imputed data are broadly similar to those fitted to non-imputed data. Only small differences in effect size or p-values were observed when predicting performance on MRCP Part 1. No overall changes in statistical significance were observed. For MRCP Part 2 performance, no differences were observed in the predictive validity of BMAT subtest scores. There are some differences in how the UCAT subtest scores behave, however. The a*bstract reasoning* subtest score becomes a non-statistically significant predictor, and the *quantitative reasoning* and total UCAT become statistically significant predictors ($p<0.05$). For predicting the performance at PACES, the UCAT *decision analysis* score becomes a predictor of only borderline statistical significance, and the total UCAT score becomes statistically significant. Again, negligible differences were observed in relation to the predictive validity of BMAT subtest scores.

DISCUSSION

In this study, we observed that both BMAT and UCAT scores have incremental predictive validity for performance on the MRCP. As might be expected, scores on different sections of the admissions tests predict performance on the various sections of the MRCP. Performance on the BMAT *aptitude and skills* subtest and on the UCAT *verbal reasoning* subtest predicted the odds of passing all sections of the MRCP at first attempt, independently of prior educational achievement.

For peer review only - http://bmjopen.bmj.com/site/about/guidelines.xhtml

Of particular interest was the relationship between the selection assessment scores and performance on the PACES, as an assessment of practical clinical skills. In those graduates who had originally sat both BMAT and the UCAT, we observed that scores on BMAT *aptitude and skills* (OR 1.34, 1.08 to 1.67) and the UCAT *verbal reasoning* (OR 1.34, 1.04 to 1.71) subtests were incrementally predictive of passing the PACES at the first attempt. We also noted that the summed scores, across the first two BMAT subtests, predicted performance on all sections of the MRCP. It was also observed that a 'rebalanced' UCAT summary score, which addresses the substantial weight on non-verbal reasoning, appears to add value over using the usual summary, total UCAT score. This was especially evident when predicting performance on the PACES, where only the 'rebalanced' total was incrementally predictive.

Consistently, BMAT *aptitude and skills* and UCAT *verbal reasoning* subtest scores showed predictive validity when considering all the MRCP related outcomes of interest. This is in line with findings from a previous study of the predictive validity of the UCAT scores, where performance on the *verbal reasoning* subtest had the strongest relationship with academic achievement throughout medical school study [13]. As with the present findings, this predictive ability was incremental over and above that provided by prior educational attainment at secondary (high) school. Thus, it is reasonable to infer that these assessment sections evaluate abilities that are both relevant to future performance in clinical tests of both pure and applied semantic knowledge and procedural skills. Moreover, at least to some extent, they must measure traits or abilities not fully covered by the, largely science-based, subjects taken at secondary school, especially at Advanced level. Previous research has shown that the BMAT *aptitude and skills* subtest can be conceptualised as measuring 'thinking skills' [17], that is, problem solving ability. The UCAT *verbal reasoning* subtest, as mentioned earlier, evaluates the ability to make inferences and draw conclusions from written information, and can be

For peer review only - http://bmjopen.bmj.com/site/about/guidelines.xhtml

considered related, though somewhat distinct from, problem solving that does not heavily rely on verbal comprehension skills [30]. Thus, both selection assessment subtests test cognitive, problem solving skills, rather than semantic, factual recall, though verbal reasoning does not seek to assess such non-verbal skills. Nevertheless, both subtests generated scores with a similar ability to predict subsequent performance in PACES.

Our findings can be considered alongside results from a study assessing the relationship between BMAT and UCAT scores and performance on the Membership of the Royal College of Surgeons (MRCS) examination. [22] In this instance, using data from a comparable time period, the authors observed a statistically significant relationship between admissions test scores and performance on the written component of the MRCS, and evidence of *aptitude and skills* and *abstract reasoning* scores incrementally predicting performance on the clinical examination component. In more recent data, after the introduction of A* grades at A-level in 2010, these relationships with the clinical assessment were not reported as statistically significant. However, the lack of observed, statistically significant, associations between selection assessment scores and clinical exam performance may have been due to a lack of study power, as only a relatively small number of individuals had sat the MRCS in the post-2010 cohort. Also, it is not reported how this study of MRCS performance controlled for the fact that some candidates had sat one of the admissions tests, while others had sat both. Thus, comparisons across the two admissions tests within that study, and indeed direct comparisons with this study, are difficult to make. Nevertheless, taken together, it appears there is some emerging evidence regarding the relationship between admissions test scores and performance on postgraduate clinical assessments more broadly. In both this study and the MRCS study, it is the scores on the section of BMAT which assesses *thinking skills* that predicts performance at the Royal College clinical assessment, rather than scores on the component of BMAT which assesses more factual recall. As discussed earlier, the UCAT

For peer review only - http://bmjopen.bmj.com/site/about/guidelines.xhtml

does not contain a component which assesses such semantic recall of knowledge, and rather aims to evaluate a number of different cognitive domains. The UCAT scale scores which possess predictive power for the clinical assessment differs between the MRCP, where *verbal reasoning* scores are statistically significant, and the MRCS, where it is the *abstract reasoning* scores. Such a difference may by explained simply by the different skills required to pass a surgical clinical assessment and those needed for a more general internal medicine clinical assessment. However, taking all the results together, it suggests that it is indeed cognitive, problem solving skills which are particularly important for predicting performance at Royal College clinical assessments, rather than semantic, factual recall.

There are some advanced school qualifications, such as ‘thinking skills’ [33] that may evaluate such cognitive, problem solving skills, though they are not generally accepted as part of entry requirements by UK medical schools. However, it may be that the high school maths and science exams, generally taken by medical school applicants, do not always test these abilities fully, typically placing more emphasis on testing non-verbal problem-solving skills and semantic factual recall. Also, as previously illustrated via modelling studies, academic or educational performance is not simply a direct result of intellectual (cognitive) ability, but can mediated by factors such as secondary school attended [34] or personality traits such as conscientiousness [35 36]. Indeed, more recently there have been attempts to identify the genetic basis of ‘non-cognitive’ abilities by partitioning education achievement from intellectual ability [37]. Moreover, when predicting performance at ‘high fidelity’ clinical assessments, factors related to inter-personal functioning, may be stronger predictors than levels of relevant semantic knowledge [38]. This is especially interesting in the case of verbal ability. Indeed, the earliest attempts to measure ‘social intelligence’ found it difficult to delineate between verbal ability and experimental measures of interpersonal competence [39]. Individuals are often judged in regards to their ‘social skills’ on the basis of the language they use, as

For peer review only - http://bmjopen.bmj.com/site/about/guidelines.xhtml

much as their behaviour. Thus, in this case, to some extent, verbal reasoning could be an indicator of interpersonal skills that would be important in performing well in tests that involved simulated patients or clinical situations. This speculation raises an obvious question about the lack of relationship observed between the BMAT *writing task* section and MRCP performance. There are several, non-mutual explanations for this. Firstly, the BMAT writing task relies on human raters making a judgment about the writing style and quality of critical thought on an essay completed within 30 minutes. Thus, the granularity of the measure and reliability would be expected to be much lower than for the selection assessment subtest with multiple items. Additionally, reliability, though no doubt quality assured, would still rest on inter-rater agreement, rather than whether a response was 'correct' or not, as with the other subtests. Thus, the scores generated from such a task may be less able to precisely differentiate between test-takers and thus be unable to predict later performance at PACES, especially in a group of generally well performing candidates. It may also be the case that the ability to argue a fluent and coherent case will be more heavily influenced by the home and school environment of a medical school applicant, than say, a test of verbal reasoning. These influences seem to be less relevant as medical education progresses and they become more distal [34]. Therefore, achievement at such an assessment taken prior to university admission may be less likely to be associated with performance in postgraduate clinical exams more than six years later.

A counterintuitive result observed was that for a higher UCAT *abstract reasoning* score to be associated with a lower odds of passing MRCP Part 2 at the first attempt. However, once missing prior educational data were imputed the abstract reasoning scores became a non-statistically significant predictor of MRCP Part 2 performance. This suggests that caution should be used when interpreting the former result. Additionally, scores on the UCAT

For peer review only - http://bmjopen.bmj.com/site/about/guidelines.xhtml

decision analysis subtest, as well as UCAT total score, became significant predictors of PACES performance in the imputed dataset.

Adjusting for A-level performance alone had very little impact on the results. This is why only the findings adjusted for both A-level and GCSE performance were presented. This is almost certainly due to the lack of variation in A-level performance for the final sample of doctors included in the analysis. Given the entry requirements for medicine, the range of prior educational performance would already be restricted. For example, over 70% of medical graduates with A-level data from before 2010 achieved the maximum available grade of AAA. This lack of observed variation would have been further constrained in the final sample of doctors sitting the MRCP relatively early in their careers, who may have been particularly academically well performing. Adjusting for GCSE performance, where the exams had been taken around the age of 16 years, had a somewhat greater impact on the adjusted results, particularly when predicting performance on Part 1 of the MRCP. This increased impact of adjusting for GCSE, rather than A-level performance, is likely due to greater variation in grades across the cohort. For future analyses it should be noted that, from 2010 onwards, the introduction of an A* grade at A-levels will have increased the variability of the grades obtained by medical students and doctors.

For peer review only

Strengths and potential limitations

We restricted our analytic sample to those individuals who sat both BMAT and UCAT. This allowed for comparison of results across the two admissions tests. However, by restricting the analysis in this way there is a risk that our results may not generalise to those individuals who sat just one of the admissions tests. As could be seen, the cohort analysed here had generally higher academic attainment than the wider cohort of medical graduates available within the UKMED. In practice, due to the relatively low proportion of medical schools using BMAT,

For peer review only - http://bmjopen.bmj.com/site/about/guidelines.xhtml

compared to the UCAT in the UK, currently relatively few medical applicants in the UK sit only BMAT, but a much greater number of applicants sit only the UCAT. As such, our results are likely to be generalisable to the wider cohort of all applicants who sit BMAT. However, the extent to which our findings would generalise to all applicants who sit the UCAT is uncertain. A number of non-statistically significant trends were noted between selection assessment scores and performance on membership exam components. Due to the stage of maturity of the cohorts being followed via the UKMED the numbers of individuals having sat the MRCP at least once were relatively low. This may mean the study was underpowered to detect relatively small effect sizes. Nevertheless, it is doubtful whether such modest effects would be of 'educational significance', even if they were found to be statistically significant. However, our analyses could be replicated at a later stage when more data are available relating to postgraduate clinical examination performance with the UKMED.

The generalisability of these results to other Royal College examinations is also unclear. Although a substantial proportion of doctors sit the MRCP as part of medical training they may not be entirely representative of all those in postgraduate training. However, broadly similar results have been observed in relation to performance on the MRCS examination [22] as observed in this study, although, as discussed earlier, caution should be exercised in making direct comparisons between the studies. Both studies used data from the UKMED. As more data become available via the UKMED the presence of similar relationships between selection test scores and other Royal College examinations could be evaluated for. As different UCAT scale scores predicted performance on the MRCP clinical assessment than on the MRCS clinical assessment, it would be important to understand how each cognitive scale of the UCAT relates to performance on a range of Royal College examinations. Moreover, those in the present sample generally sat the exam relatively early in their careers, and as

For peer review only - http://bmjopen.bmj.com/site/about/guidelines.xhtml

UKMED continues to mature, a sample including those who sat the MRCP relatively later in their training could additionally be evaluated.

The selection assessment scores were standardised using cohort means and standard deviations. This was the most appropriate method of including scores in analyses across years. However, some limitations of this approach should be acknowledged. The approach assumes that the ability of the population of test takers (on any test construct) is stable year on year. This may not always be the case, partly as the schools which required each test for admission has changed over time. However, such an adjustment was necessary as non-standardised scores would be rendered even less comparable across cohorts. For example, secular trends may be at work. For example, as selection tests have become widely used and established, applicants may have spent more time developing the skills tested by these exams, so later cohorts are likely to have higher ability, on average, compared to previous ones. Our approach to standardisation may have helped address such temporal influences.

Lastly, the rationale for our study mainly rests on the premise that postgraduate clinical assessments may be a proxy for real world performance. Ideally admissions scores would be linked to metrics of actual clinical performance. However, this is extremely challenging in practise. It may be considered that competent performance in a clinical assessment is perhaps a necessary, though not sufficient, condition for actual workplace practise. In this regard it has been reported that in non-UK medical graduates, increased performance in the practical component of a licensing examination was protective of the risk of future fitness to practise events [40].

Potential policy implications

When considering evidence for the validity of an assessment it is sometimes useful to apply Kane's validity framework [41]. This is an argument-based approach, whereby a critique of an

For peer review only - http://bmjopen.bmj.com/site/about/guidelines.xhtml

assessment is placed in the context of the claims made for the test, as well as how the resulting test scores are used in practice. In this regard the claims made for the UCAT are more ambitious than those of the developers of BMAT. Specifically the UCAT developers claim that the assessment is *'seeking to identify the characteristics in applicants which will make the good clinicians and thus to improve the quality of those who enter the professions with the ultimate aim of improving patient care.'* [42]. Our findings here suggest that the UCAT could potentially be useful in identifying medical applicants likely to become good future clinicians, if we accept the assumption that MRCP performances, and in particular performance on PACES, are a reasonable proxy for actual clinical behaviour. Testing this assumption is, as mentioned earlier, non-trivial. However, there is evidence that clinical examinations have face validity with clinicians [43], which suggests this assumption may indeed be plausible.

Nevertheless, the potential of the UCAT to improve medical selection with regards to selecting those applicants likely to make good doctors will not be fully realised unless the resultant scores are reported and used by selectors in the optimally effective way. In particular it should be noted that reweighting the summary UCAT scores into a 'rebalanced' UCAT summary score, which did not overemphasise non-verbal reasoning, appeared to provide greater effect sizes, particularly when predicting performance on Part 2 and the PACES component of the MRCP. Selectors themselves may also wish to consider placing more weight on the verbal reasoning component of the UCAT if they wish to realise additional incremental predictive ability for both undergraduate and postgraduate educational performance. However, some caution should be exercised with this approach; previously it was reported that verbal reasoning was the UCAT cognitive subtest score that was most sensitive to socioeconomic status in medical school applicants [7].

For peer review only - http://bmjopen.bmj.com/site/about/guidelines.xhtml

In contrast to the UCAT, BMAT claims, less ambitiously, that the assessment is intended to identify those with "*...potential to succeed on medical and health-related courses*." [14]. In this sense, the present findings largely support this claim, though performance on the writing task section seemed to have little relationship with postgraduate performance in this sample. Moreover, despite the modest claimed aspirations, BMAT component scores and summed scores did seem to have some incremental predictive ability regarding future PACES performance. Unsurprisingly, BMAT scientific knowledge and applications section scores tended to predict performance on Part 1 and 2 of the MRCP, which depends on the ability to recall factual information. However, these predictions remained fairly substantial even after correcting for the influence of prior educational achievement. This suggests that such components of selection assessments may add value above secondary school qualifications, probably by providing a more fine-grained metric, which is able to differentiate test takers at the top end of ability. This is in contrast to the UK based school exams, such as A-levels and GCSEs, where many medical school applicants often achieve straight As (or, from 2010 onwards, A*s). Moreover, such semantic knowledge test may be of practical use in selection when considering applications from overseas candidates, where the equivalence of educational qualifications in the sciences may be in uncertain, or difficult to equate. Nevertheless, consideration should be given to the potential impact on the diversity and widening access agenda if knowledge tests are to be included in the selection process. Such tests are likely to be more sensitive to sociodemographic background factors and coaching effects compared to measures of problem-solving ability. However, it should be noted that, assessments of the latter are also prone, to some extent, to practise and coaching effects [44]. Moreover, if the overall goal of medical schools is to produce clinically competent doctors, rather than students and trainees who merely do well at exams requiring factual recall, then

For peer review only - http://bmjopen.bmj.com/site/about/guidelines.xhtml

more emphasis on the scores from assessments evaluating problem solving ability may be advisable.

BMAT and UCAT have been mainly used in the UK. Recently the UCAT has been introduced into medical selection by some universities in Australasia. Moreover, the use of such selection assessments, which often have components evaluated both semantic knowledge, and problem-solving skills, are increasingly being used globally. Consequently, our findings have implications for medical selection internationally. The MCAT, used widely across North America, is one such test. Scores from the MCAT have been shown to predict USMLE performance.[27] No equivalent to the USMLE currently exists in the UK medical education system. However, it is generally sat much earlier than the Royal College examinations in the respective training pathways. Royal College examinations in the UK are more similar to board certification examinations in the US. Perhaps the closest equivalent to the MRCP in the US context is the American Board of Internal Medicine certification examination.[45] That scores from both BMAT and UCAT can predict such distal outcomes adds to the international evidence supporting the use of admissions testing. Furthermore, those sections which assess problem solving or verbal reasoning add most value to the selection process for clinical assessment performance, which we assume to be a reasonable proxy for actual clinical practice. As many admissions tests in use around the world assess such abilities, including the MCAT, our findings may have implications for medical selection internationally, although we note that the primary outcome considered here (high-fidelity clinical assessment performance) is perhaps less established in the US than in other Western medical training settings.[46] That being said, research should be undertaken to determine if this indeed is the case in each context locally.

Closer to home, in the UK, undergraduate medical admissions teams are generally faced with a choice of either BMAT or UCAT as the selection assessment to employ. The present

For peer review only - http://bmjopen.bmj.com/site/about/guidelines.xhtml

findings do not highlight any clear advantage, in terms of predictive validity, of one over the other. Therefore, choices should be informed by other test qualities, such as the potential impact on widening access and diversifying medicine. In this sense, both BMAT and UCAT have components where performance is associated with certain candidate background factors [7 47]. Thus, at present, in this respect, there would seem to be no overall clear advantage of one selection assessment over the other.

Directions for future research

In addition to replicating these findings in other postgraduate exams and subsequent cohorts of medical graduates, there are future opportunities, provided by UKMED, to evaluate the validity of medical selection assessments. In particular, an SJT was added to the UCAT in 2013. Previous research has shown that scores on the SJT do possess some predictive validity for undergraduate supervisor ratings [48]. In this context, it will be important to consider to what extent the SJT scores predict performance in clinical assessments, and other construct-relevant outcomes, and whether this is incremental to other selection measures.

Another change made to the structure of the UCAT has been the replacement of the decision analysis subtest for ‘decision making’ in 2017. Once the data availability allows, it would be important to estimate the relationship between *decision making* and the performance on PACES and other postgraduate practical examinations.

Given that the two selection assessments considered here had comparable predictive validity, as mentioned above, other properties should be considered when choosing such a test for use in medical admissions processes. Specifically, the potential impact on the diversity of the selected population should be considered. It has been highlighted that the trade-off between selecting for future performance and ‘adverse impact’ on certain underrepresented groups can be conceptualised, and modelled, as a problem of ‘pareto-optimisation’ [49]. That is, there are

For peer review only - http://bmjopen.bmj.com/site/about/guidelines.xhtml

optimal, and suboptimal, combinations of how elements within a selection process can be weighted and combined to provide better and worse trade-offs in this regard. The sensitivity of both BMAT and UCAT scores to a variety of sociodemographic factors have been previously described. Scores on BMAT for applicants to one medical school displayed sensitivity to gender, secondary school type attended, and some measures of socioeconomic status [47]. Similarly, scores on the UCAT, including the SJT, display sensitivity to gender, socioeconomic status, age, ethnicity and secondary school type attended [7 50]. These studies are not directly comparable, analysing somewhat different background variables and cohorts. Nevertheless, taken together, the findings suggest that both BMAT and UCAT scores display some sensitivity to some sociodemographic characteristics. Such information is vital if selectors are to make informed choices, not only about the tools they employ, but how different elements of the selection metrics are combined and weighted in order to achieve 'pareto-optimality'; that is, improvement in one dimension without degrading another.

Ultimately, linking selection test scores to relevant patient outcomes in clinical practice would be the most desirable source of validity evidence. This would shed light on how, if at all, such assessment scores could predict typical performance, in contrast to the maximal performance observed in high stakes postgraduate clinical exams. Nevertheless, as emphasised early, this is extremely challenging, though more advanced statistical techniques may eventually be able to disentangle environment from individual physician effects. Such approaches would depend on clinicians working across different (ideally small) teams in the hope that the individual doctor's clinical 'footprint' might be observable.

Conclusions

Our findings suggest that both BMAT and UCAT contain components that show some incremental predictive validity for MRCP performance, with broadly similar effect sizes. The

For peer review only - http://bmjopen.bmj.com/site/about/guidelines.xhtml

subtests which assess the constructs of ‘thinking skills’ and ‘verbal reasoning’ appeared of most value, over and above conventional metrics of educational performance, in this regard. Thus, it could be argued it is these aspects of cognitive performance that may be of most value in the selection of future doctors in this context. Both test developers and selectors should consider this evidence when considering how such assessments should be constructed and used within the medical selection process.

LIST OF ABBREVIATIONS

- BMAT: BioMedical Admissions Test
- GCSE: General Certificate of Secondary Education
- MCAT: Medical College Admissions Test
- MRCP: Membership of the Royal Colleges of Physicians of the UK
- MRCS: Membership of the Royal College of Surgeons
- PACES: Practical Assessment of Clinical Examination Skills
- SJT: Situational Judgement Test
- UCAT: Universities Clinical Aptitude Test
- UKMED: UK Medical Education Database
- USMLE: US Medical Licensing Examination

DECLARATIONS

Ethics approval and consent to participate

Ethical approval for the study was not required as it relied on the analysis of de-identified routinely collected data. This was confirmed in writing by the chair of the University of York’s Health Sciences Ethics committee.

Data sharing statement

For peer review only - http://bmjopen.bmj.com/site/about/guidelines.xhtml

The dataset supporting the conclusions of this article is available from UKMED on application (www.ukmed.ac.uk).

UK Medical Education Database ("UKMED") P051 extract generated on 13/05/2019. Approved for publication on 20/11/2019. We are grateful to UKMED for the use of these data. However, UKMED bears no responsibility for their analysis or interpretation. The data includes information derived from that collected by the Higher Education Statistics Agency Limited ("HESA") and provided to the GMC ("HESA Data"). Source: HESA Student Record 2002/03 to 2017/18 Copyright Higher Education Statistics Agency Limited. The Higher Education Statistics Agency Limited makes no warranty as to the accuracy of the HESA Data, cannot accept responsibility for any inferences or conclusions derived by third parties from data or other information supplied by it.

Competing interests

UCAT pay for a portion of LWP's research time. LWP and PAT have received travel expenses for attendance at UCAT consortium meetings. PAT has previously received research funding from the UCAT consortium. KYFC is an employee of Cambridge Assessment - a group of exam boards that owns and administers the BioMedical Admissions Test (BMAT); UK GCSEs and A-levels; and International GCSEs and A-levels. ICM is member of various MRCP(UK) committees overseeing and analysing results from MRCP(UK) examinations.

Funding

LWP is partly funded by the UCAT Consortium. The UCAT consortium partly funded this research but did not play an active role in determining the study design or reporting the results.

For peer review only - http://bmjopen.bmj.com/site/about/guidelines.xhtml

University College London receives money from MRCP(UK) for the contribution of ICM to the running of the examination, but ICM receives none of that money.

DS is employed by the GMC as a data analyst working on the UKMED project. The views expressed here are his views and not the views of the GMC.

PAT's research time for this project was funded by an NIHR Career Development Fellowship (CDF-2015-08-11). This paper presents independent research funded by the National Institute for Health Research (NIHR). The views expressed are those of the authors and not necessarily those of the NHS, the NIHR or the Department of Health and Social Care.

Authors contributions

PAT led on conception of the project, with support from all authors. LWP led on data analysis with support from PAT. All authors contributed to interpretation of the results. LWP led on writing of the manuscript, with support from all authors. All authors have approved the final version of the article submitted.

Acknowledgements

We would like to thank Rachel Greatrix at UCAT for her comments on an earlier version of this manuscript.

REFERENCES

1. McManus I, Woolf K, Dacre J, et al. The Academic Backbone: Longitudinal Continuities in Educational Achievement from Secondary School and Medical School to MRCP(UK) and the Specialist Register in UK Medical Students and Doctors. *BMC Medicine* 2013;11:242.

For peer review only - http://bmjopen.bmj.com/site/about/guidelines.xhtml

2. Mercer A, Puddey IB. Admission selection criteria as predictors of outcomes in an undergraduate medical course: a prospective study. *Medical Teacher* 2011;33(12):997-1004.

3. Kim T, Chang J-Y, Myung SJ, et al. Predictors of undergraduate and postgraduate clinical performance: a longitudinal cohort study. *Journal of Surgical Education* 2016;73(4):715-20.

4. Al Alwan I, Al Kushi M, Tamim H, et al. Health sciences and medical college preadmission criteria and prediction of in-course academic performance: a longitudinal cohort study. *Advances in Health Sciences Education* 2013;18(3):427-38.

5. Shulruf B, Poole P, Wang GY, et al. How well do selection tools predict performance later in a medical programme? *Advances in Health Sciences Education* 2012;17(5):615-26.

6. Gupta N, Nagpal G, Dhaliwal U. Student performance during the medical course: role of pre-admission eligibility and selection criteria. *Natl Med J India* 2013;26(4):223-6.

7. Tiffin PA, McLachlan JC, Webster LAD, et al. Comparison of the sensitivity of the UKCAT and A Levels to sociodemographic characteristics: a national study. *BMC Medical Education* 2014;14:7.

8. Emery JL, Bell JF. The predictive validity of the BioMedical Admissions Test for pre-clinical examination performance. *Medical education* 2009;43(6):557-64.

9. Hissbach JC, Klusmann D, Hampe W. Dimensionality and predictive validity of the HAM-Nat, a test of natural sciences for medical school admission. *BMC Medical Education* 2011;11(1):83.

10. Kelly ME, Regan D, Dunne F, et al. To what extent does the Health Professions Admission Test-Ireland predict performance in early undergraduate tests of communication and clinical skills?–An observational cohort study. *BMC Medical Education* 2013;13(1):68.

For peer review only - http://bmjopen.bmj.com/site/about/guidelines.xhtml

11. Julian ER. Validity of the Medical College Admission Test for predicting medical school performance. *Academic Medicine* 2005;80(10):910-17.

12. Poole P, Shulruf B, Rudland J, et al. Comparison of UMAT scores and GPA in prediction of performance in medical school: a national study. *Medical Education* 2012;46(2):163-71.

13. Tiffin PA, Mwandigha LM, Paton LW, et al. Predictive validity of the UKCAT for medical school undergraduate performance: a national prospective cohort study. *BMC Medicine* 2016;14(1):140.

14. Cambridge Assessment Admissions Testing. The BioMedical Admissions Test (BMAT) [Available from: http://www.admissionstestingservice.org/for-test-takers/bmat/ accessed 12th February 2021.

15. UCAT. About UCAT [Available from: https://www.ucat.ac.uk/about-ucat/ accessed 12th February 2021.

16. Medical Schools Council. Entry Requirements [Available from: https://www.medschools.ac.uk/studying-medicine/making-an-application/entry-requirements accessed 24th February 2021.

17. Cheung KYF, McElwee S. What skills are we assessing? Cognitive validity in BMAT. In: Cheung KYF, McElwee S, Emery J, eds. Applying the socio-cognitive framework to the BioMedical Admissions Test (BMAT): Insights from language assessment. Cambridge, UK: Cambridge University Press 2017:35-80.

18. UCAT. UCAT Test Format [Available from: https://www.ucat.ac.uk/ucat/test-format/.

19. McManus I, Dewberry C, Nicholson S, et al. The UKCAT-12 Study: Educational Attainment, Aptitude Test Performance, Demographic and Socio-Economic Contextual Factors as Predictors of First Year Outcome in a Collaborative Study of Twelve UK Medical Schools. *BMC Medicine* 2013;11:244.

For peer review only - http://bmjopen.bmj.com/site/about/guidelines.xhtml

20. MacKenzie R, Cleland J, Ayansina D, et al. Does the UKCAT predict performance on exit from medical school? A national cohort study. *BMJ open* 2016;6(10):e011313.

21. McManus IC, Woolf K, Harrison D, et al. Calculated grades, predicted grades, forecasted grades and actual A-level grades: Reliability, correlations and predictive validity in medical school applicants, undergraduates, and postgraduates in a time of COVID-19. *medRxiv* 2020

22. Ellis R, Brennan P, Scrimgeour DS, et al. Performance at medical school selection correlates with success in Part A of the intercollegiate Membership of the Royal College of Surgeons (MRCS) examination. *Postgraduate Medical Journal* 2021

23. Royal Colleges of Physicians of the United Kingdom. MRCPUK [Available from: https://www.mrcpuk.org/.

24. MRCP(UK). Exam pass marks [Available from: https://www.mrcpuk.org/mrcpuk-examinations/results/exam-pass-marks accessed 10th June 2020.

25. Dowell J, Cleland J, Fitzpatrick S, et al. The UK medical education database (UKMED) what is it? Why and how might you use it? *BMC Medical Education* 2018;18(1):6. doi: 10.1186/s12909-017-1115-9

26. Donnon T, Paolucci EO, Violato C. The predictive validity of the MCAT for medical school performance and medical board licensing examinations: a meta-analysis of the published research. *Academic Medicine* 2007;82(1):100-06.

27. Gauer JL, Wolff JM, Jackson JB. Do MCAT scores predict USMLE scores? An analysis on 5 years of medical student data. *Medical Education Online* 2016;21(1):31795.

28. UK Medical Education Database. Process for completing UKMED research - version 4 2019 [Available from: https://www.ukmed.ac.uk/documents/UKMED_research_process.pdf.

For peer review only - http://bmjopen.bmj.com/site/about/guidelines.xhtml

29. McManus I, Ludka K. Resitting a high-stakes postgraduate medical examination on multiple occasions: nonlinear multilevel modelling of performance in the MRCP(UK) examinations. *BMC Medicine* 2012;10:60.

30. Tiffin PA. Understanding the Dimensionality and Reliability of the Cognitive Scales of the UK Clinical Aptitude test (UKCAT): Summary Version of the Report 2013 [Available from: https://www.ucat.ac.uk/media/1182/understanding-the-dimensionality-and-reliability-of-the-cognitive-scales-of-the-ukcat.pdf accessed 25 June 2017 2017.

31. McManus I, Elder A, De Champlain A, et al. Graduates of different UK medical schools show substantial differences in performance on MRCP(UK) Part 1, Part 2 and PACES examinations. *BMC Medicine* 2008;6:5.

32. Stata 14 for Windows [program]. College Station, TX: StataCorp.

33. Cambridge Assessment International Education. Cambridge International AS and A Level Thinking Skills (9694) [Available from: https://www.cambridgeinternational.org/programmes-and-qualifications/cambridge-international-as-and-a-level-thinking-skills-9694/ accessed 1st March 2021.

34. Mwandigha LM, Tiffin PA, Paton LW, et al. What is the effect of secondary (high) schooling on subsequent medical school performance? A national, UK-based, cohort study. *BMJ Open* 2018;8(5) doi: 10.1136/bmjopen-2017-020291

35. Tiffin PA, Finn GM, McLachlan JC. Evaluating professionalism in medical undergraduates using selected response questions: findings from an item response modelling study. *BMC Medical Education* 2011;11:43.

36. Conrad N, Patry M. Conscientiousness and Academic Performance: A Mediational Analysis. *International Journal for the Scholarship of Teaching and Learning* 2012;6 doi: 10.20429/ijsotl.2012.060108

For peer review only - http://bmjopen.bmj.com/site/about/guidelines.xhtml

37. Demange PA, Malanchini M, Mallard TT, et al. Investigating the genetic architecture of noncognitive skills using GWAS-by-subtraction. *Nature Genetics* 2021;53(1):35-44. doi: 10.1038/s41588-020-00754-2

38. Patterson F, Tiffin PA, Lopes S, et al. Unpacking the dark variance of differential attainment on examinations in overseas graduates. *Medical Education* 2018;52(7):736-46. doi: doi:10.1111/medu.13605

39. Matthews G, Zeidner M, Roberts R. Appendix A: A Review and Critique of Social Intelligence. Emotiional Intelligence: Science and Myth. Cambridge, MA: The MIT Press 2000:551-61.

40. Tiffin PA, Paton LW, Mwandigha LM, et al. Predicting fitness to practise events in international medical graduates who registered as UK doctors via the Professional and Linguistic Assessments Board (PLAB) system: a national cohort study. *BMC Medicine* 2017;15(1):66. doi: 10.1186/s12916-017-0829-1

41. Kane MT. Validating the Interpretations and Uses of Test Scores. *Journal of Educational Measurement* 2013;50(1):1-73.

42. UCAT. University Clinical Aptitude Test [Available from: https://www.ucat.ac.uk/ accessed 12th February 2021.

43. Elder AT, McManus IC, Patrick A, et al. The value of the physical examination in clinical practice: an international survey. *Clinical Medicine* 2017;17(6):490.

44. Hausknecht JP, Halpert JA, Di Paolo NT, et al. Retesting in selection: a meta-analysis of coaching and practice effects for tests of cognitive ability. *Journal of Applied Psychology* 2007;92(2):373.

45. American Board of Internal Medicine. Becoming Certified [Available from: https://www.abim.org/certification/default.aspx accessed 19th March 2021.

For peer review only - http://bmjopen.bmj.com/site/about/guidelines.xhtml

46. Elder A, Chi J, Ozdalga E, et al. The road back to the bedside. *JAMA* 2020;323(17):1672-73.

47. Emery JL, Bell JF, Vidal Rodeiro CL. The BioMedical admissions test for medical student selection: issues of fairness and bias. *Medical Teacher* 2011;33(1):62-71.

48. Patterson F, Cousans F, Edwards H, et al. The Predictive Validity of a Text-Based Situational Judgment Test in Undergraduate Medical and Dental School Admissions. *Academic Medicine* 2017 doi: 10.1097/acm.0000000000001630

49. De Corte W, Sackett P, Lievens F. Designing Pareto-Optimal Selection Systems: Formalizing the Decisions Required for Selection System Development. *Journal of Applied Psychology* 2011;96:907-26.

50. Lievens F, Patterson F, Corstjens J, et al. Widening access in selection using situational judgement tests: evidence from the UKCAT. *Medical Education* 2016;50(6):624-36.

For peer review only - http://bmjopen.bmj.com/site/about/guidelines.xhtml

TABLES

	All graduates (n=69,885 in total, incl. 20,175 individuals who sat BMAT and 43,685 individuals who sat UCAT)	Entrants who sat both BMAT and UCAT (n=13,505)	Individuals who sat BMAT, UCAT and had data available for at least one section of the MRCP (n=3,045)	Individuals who sat BMAT, UCAT, MRCP, had complete prior educational data and sat A-levels prior to 2010 (n=1,320)
**Age at BMAT**	17.73 (1.67)	17.68 (1.47)	17.62 (1.31)	17.46 (0.92)
**Age at UCAT**	18.59 (2.91)	17.74 (1.54)	17.70 (1.39)	17.47 (0.94)
**Sex**	43.02% male	47.04% male	48.05% male	48.33% male
**BMAT: AaS**	5.18 (1.06)	5.19 (1.13)	5.28 (1.15)	5.28 (1.10)
**BMAT: SKaA**	5.09 (1.00)	5.13 (1.05)	5.24 (1.08)	5.25 (1.04)
**BMAT: W**	8.92 (2.32)	9.20 (2.30)	8.51 (1.98)	8.49 (1.89)
**UCAT: AR**	636.76 (82.36)	648.91 (81.13)	644.42 (80.21)	647.15 (79.41)
**UCAT: DA**	659.44 (89.34)	673.06 (86.54)	666.82 (89.17)	673.58 (88.52)
**UCAT: QR**	668.82 (81.49)	684.76 (80.86)	677.42 (77.87)	687.53 (70.17)
**UCAT: VR**	617.81 (76.48)	626.37 (76.05)	635.07 (78.04)	637.96 (73.33)
**A-level performance, standardised by applicants**	0.04 (0.98)	0.25 (0.79)	0.27 (0.71)	0.20 (0.73)
**GCSE performance, standardised by applicants**	0.29 (0.83)	0.47 (0.80)	0.55 (0.76)	0.65 (0.58)

Table 1. Descriptive statistics across various sub-cohorts of the dataset. Means and standard deviations are presented, except for *sex* where percentages are displayed. AaS = aptitude and skills; SKaA = scientific knowledge and applications; W = writing; AR = abstract reasoning; DA = decision analysis; QR = quantitative reasoning; VR = verbal reasoning.

FIGURE LEGENDS

For peer review only - http://bmjopen.bmj.com/site/about/guidelines.xhtml

Figure 1. The flow of data through this study.

Figure 2. Predictive validity of each standardised section score of BMAT for passing each section of the MRCP at the first attempt. Results are shown both unadjusted and adjusted for prior educational performance. AaS = aptitude and skills; SKaA = scientific knowledge and applications; W = writing task.

Figure 3. Predictive validity of each standardised section score of the UCAT for passing each section of the MRCP at the first attempt. Results are shown both unadjusted and adjusted for prior educational performance. AR = abstract reasoning; DA = decision analysis; QR = quantitative reasoning; VR = verbal reasoning.

For peer review only

For peer review only - http://bmjopen.bmj.com/site/about/guidelines.xhtml

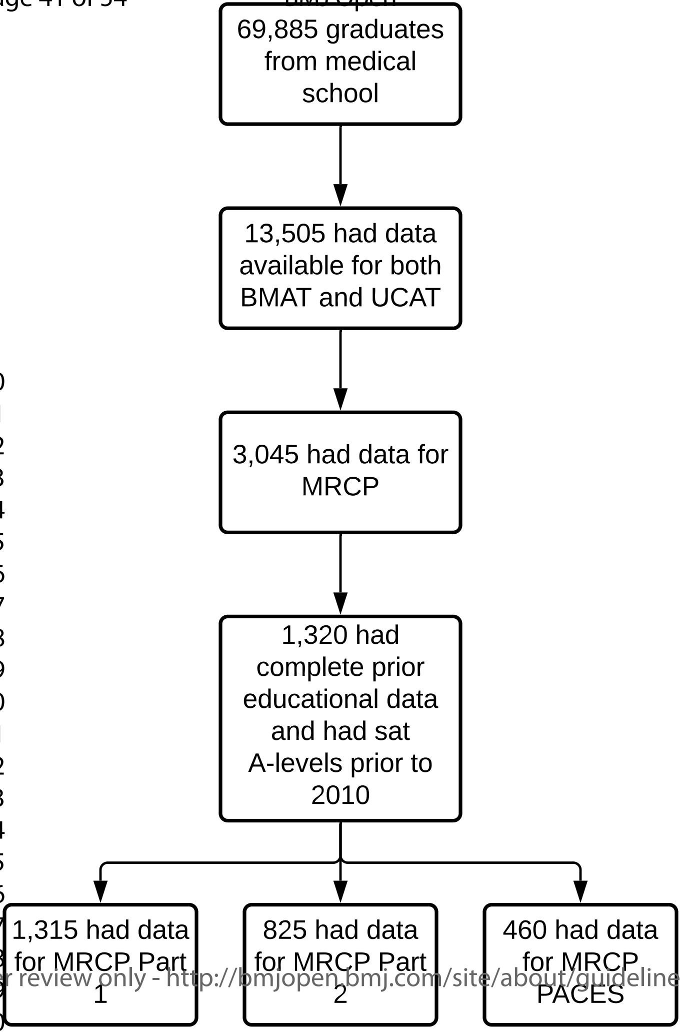

peer review only - http://bmjopen.bmj.com/site/about/guidelines.xh

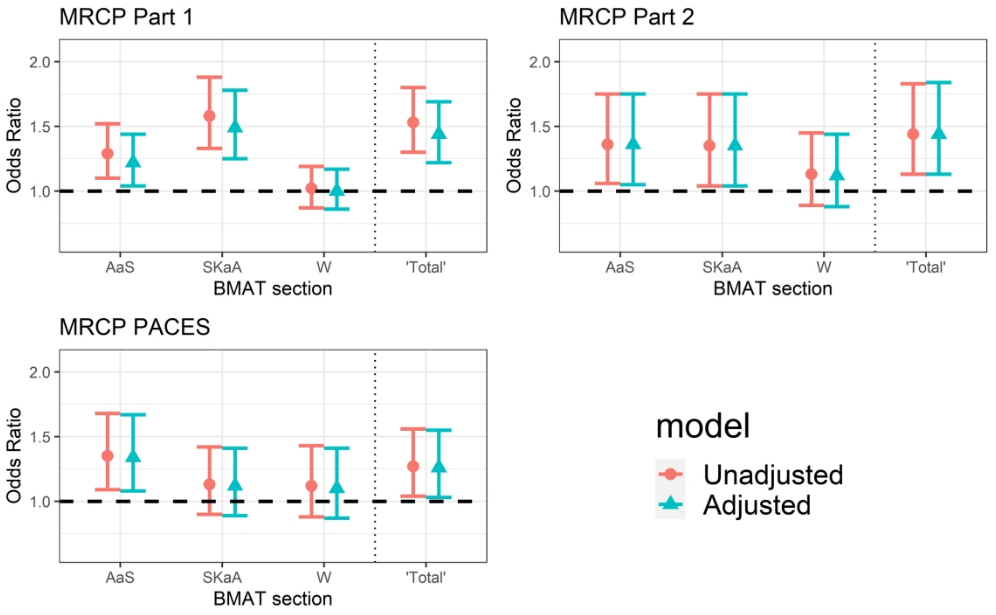

Figure 2. Predictive validity of each standardised section score of BMAT for passing each section of the MRCP at the first attempt. Results are shown both unadjusted and adjusted for prior educational performance. AaS = aptitude and skills; SKaA = scientific knowledge and applications; W = writing task.

202x125mm (300 x 300 DPI)

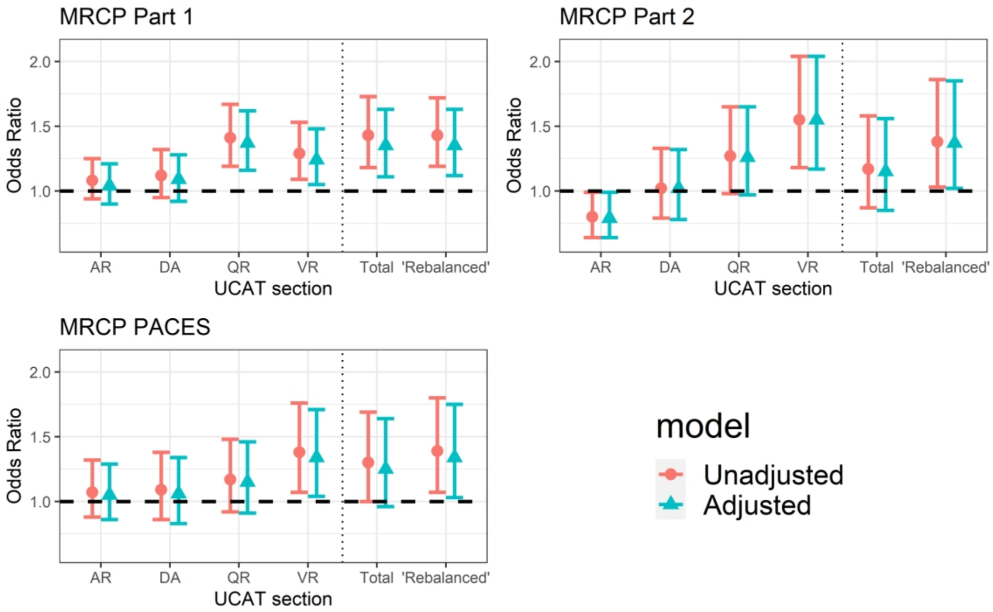

Figure 3. Predictive validity of each standardised section score of the UCAT for passing each section of the MRCP at the first attempt. Results are shown both unadjusted and adjusted for prior educational performance. AR = abstract reasoning; DA = decision analysis; QR = quantitative reasoning; VR = verbal reasoning.

202x125mm (300 x 300 DPI)

Supplemental Material for 'Can achievement at medical admissions tests predict future performance in postgraduate clinical assessments? A national cohort study'

Lewis W Paton, I C McManus, Kevin Y F Cheung, Daniel S Smith, Paul A Tiffin

A: Passing each section of the MRCP at the first attempt

Tables A1 – A3 display the results for models predicting passing each section of the Membership of the Royal Colleges of Physicians of the UK (MRCP) at the first attempt. These results are also depicted in Figures 2 and 3.

Pass at 1st attempt	MRCP: Part 1	
	**Unadjusted ORs**	**ORs adjusted for PEA**
**BMAT**		
**Aptitude and Skills**	1.29 (1.10 to 1.52) p=0.002	1.22 (1.04 to 1.44) p=0.02
**Scientific Knowledge and Applications**	1.58 (1.33 to 1.88) p<0.001	1.49 (1.25 to 1.78) p<0.001
**Writing**	1.02 (0.87 to 1.19) p=0.84	1.00 (0.86 to 1.17) p=0.98
**'Total'**	1.53 (1.30 to 1.80) p<0.001	1.44 (1.22 to 1.69) p<0.001
**UCAT**		
**Abstract Reasoning**	1.08 (0.94 to 1.25) p=0.28	1.04 (0.90 to 1.21) p=0.56
**Decision Analysis**	1.12 (0.95 to 1.32) p=0.17	1.09 (0.92 to 1.28) p=0.32
**Quantitative Reasoning**	1.41 (1.19 to 1.67) p<0.001	1.37 (1.16 to 1.62) p<0.001
**Verbal Reasoning**	1.29 (1.09 to 1.53) p=0.003	1.24 (1.05 to 1.48) p=0.01
**Total**	1.43 (1.18 to 1.73) p<0.001	1.35 (1.11 to 1.63) p=0.003
**'Rebalanced' total**	1.43 (1.19 to 1.72) p<0.001	1.35 (1.12 to 1.63) p<0.01

Table A1. Results from multilevel logistic regression models predicting passing *MRCP: Part 1* at the first attempt, unadjusted and adjusted for prior educational attainment (PEA).

For peer review only

Pass at 1st attempt	MRCP: Part 2	
	Unadjusted ORs	ORs adjusted for PEA
**BMAT**		
**Aptitude and Skills**	1.36 (1.06 to 1.75) p=0.02	1.36 (1.05 to 1.75) p=0.02
**Scientific Knowledge and Applications**	1.35 (1.04 to 1.75) p=0.02	1.35 (1.04 to 1.75) p=0.02
**Writing**	1.13 (0.89 to 1.45) p=0.32	1.12 (0.88 to 1.44) p=0.36
**'Total'**	1.44 (1.13 to 1.83) p<0.01	1.44 (1.13 to 1.84) p<0.01
**UCAT**		
**Abstract Reasoning**	0.80 (0.64 to 0.99) p=0.04	0.79 (0.64 to 0.99) p=0.04
**Decision Analysis**	1.02 (0.79 to 1.33) p=0.86	1.02 (0.78 to 1.32) p=0.91
**Quantitative Reasoning**	1.27 (0.98 to 1.65) p=0.07	1.26 (0.97 to 1.65) p=0.08
**Verbal Reasoning**	1.55 (1.18 to 2.04) p=0.002	1.55 (1.17 to 2.04) p<0.01
**Total**	1.17 (0.87 to 1.58) p=0.30	1.15 (0.85 to 1.56) p=0.36
**'Balanced' total**	1.38 (1.03 to 1.86) p=0.03	1.37 (1.02 to 1.85) p=0.04

Table A2. Results from multilevel logistic regression models predicting passing *MRCP: Part 2* at the first attempt, unadjusted and adjusted for prior educational attainment (PEA).

For peer review only

Pass at 1st attempt	MRCP: PACES	
	Unadjusted ORs	ORs adjusted for PEA
**BMAT**		
**Aptitude and Skills**	1.35 (1.09 to 1.68) p=0.01	1.34 (1.08 to 1.67) p=0.01
**Scientific Knowledge and Applications**	1.13 (0.90 to 1.42) p=0.30	1.12 (0.89 to 1.41) p=0.34
**Writing**	1.12 (0.88 to 1.43) p=0.34	1.10 (0.87 to 1.41) p=0.42
**'Total'**	1.27 (1.04 to 1.56) p=0.02	1.26 (1.03 to 1.55) p=0.03
**UCAT**		
**Abstract Reasoning**	1.07 (0.88 to 1.32) p=0.49	1.05 (0.86 to 1.29) p=0.63
**Decision Analysis**	1.09 (0.86 to 1.38) p=0.47	1.06 (0.83 to 1.34) p=0.66
**Quantitative Reasoning**	1.17 (0.92 to 1.48) p=0.20	1.15 (0.91 to 1.46) p=0.25
**Verbal Reasoning**	1.38 (1.07 to 1.76) p=0.01	1.34 (1.04 to 1.71) p=0.02
**Total**	1.30 (>1.00 to 1.69) p=0.05	1.25 (0.96 to 1.64) p=0.10
**'Rebalanced' total**	1.39 (1.07 to 1.80) p=0.01	1.34 (1.03 to 1.75) p=0.03

Table A3. Results from multilevel logistic regression models predicting passing *MRCP: PACES* at the first attempt, unadjusted and adjusted for prior educational attainment (PEA).

For peer review only

B: Imputed results

Tables B1-B3 display results from multilevel logistic models fitted to both non-imputed and imputed data. These models are adjusted for prior educational attainment.

Pass at 1st attempt	MRCP: Part 1	
	Adjusted ORs	
	Non-imputed	Imputed
**BMAT**		
**Aptitude and skills**	1.22 (1.04 to 1.44) p=0.02	1.25 (1.08 to 1.45) p<0.01
**Scientific knowledge and applications**	1.49 (1.25 to 1.78) p<0.001	1.52 (1.30 to 1.78) p<0.001
**Writing**	1.00 (0.86 to 1.17) p=0.98	1.08 (0.94 to 1.24) p=0.30
**'Total'**	1.44 (1.22 to 1.69) p<0.001	1.48 (1.28 to 1.71) p<0.001
**UCAT**		
**Abstract Reasoning**	1.04 (0.90 to 1.21) p=0.56	1.07 (0.98 to 1.17) p=0.14
**Decision Analysis**	1.09 (0.92 to 1.28) p=0.32	1.10 (<1.00 to 1.21) p=0.06
**Quantitative Reasoning**	1.37 (1.16 to 1.62) p<0.001	1.31 (1.18 to 1.45) p<0.001
**Verbal Reasoning**	1.24 (1.05 to 1.48) p=0.01	1.30 (1.17 to 1.43) p<0.001
**Total**	1.35 (1.11 to 1.63) p=0.003	1.37 (1.22 to 1.54) p<0.001
**'Rebalanced' total**	1.35 (1.12 to 1.63) p<0.01	1.40 (1.25 to 1.56) p<0.001

Table B1. Comparison of odds ratios on non-imputed and imputed data, predicting passing *MRCP: Part 1* at the first attempt. All models are adjusted for prior educational attainment.

For peer review only

Pass at 1st attempt	MRCP: Part 2	
	Adjusted ORs	
	Non-imputed	Imputed
**BMAT**		
**Aptitude and skills**	1.36 (1.05 to 1.75) p=0.02	1.35 (1.09 to 1.67) p=0.01
**Scientific knowledge and applications**	1.35 (1.04 to 1.75) p=0.02	1.33 (1.06 to 1.65) p=0.01
**Writing**	1.12 (0.88 to 1.44) p=0.36	1.14 (0.92 to 1.40) p=0.24
**'Total'**	1.44 (1.13 to 1.84) p<0.01	1.40 (1.13 to 1.72) p<0.01
**UCAT**		
**Abstract Reasoning**	0.79 (0.64 to 0.99) p=0.04	1.00 (0.87 to 1.16) p=0.97
**Decision Analysis**	1.02 (0.78 to 1.32) p=0.91	0.98 (0.83 to 1.12) p=0.66
**Quantitative Reasoning**	1.26 (0.97 to 1.65) p=0.08	1.22 (1.04 to 1.44) p=0.02
**Verbal Reasoning**	1.55 (1.17 to 2.04) p=0.002	1.47 (1.25 to 1.74) p<0.001
**Total**	1.15 (0.85 to 1.56) p=0.36	1.26 (1.05 to 1.52) p=0.01
**'Rebalanced' total**	1.37 (1.02 to 1.85) p=0.04	1.42 (1.19 to 1.70) p<0.001

Table B2. Comparison of odds ratios on non-imputed and imputed data, predicting passing *MRCP: Part 2* at the first attempt. All models are adjusted for prior educational attainment.

For peer review only

Pass at 1st attempt	MRCP: PACES	
	Adjusted ORs	
	Non-imputed	Imputed
**BMAT**		
**Aptitude and skills**	1.34 (1.08 to 1.67) p=0.01	1.20 (1.03 to 1.40) p=0.02
**Scientific knowledge and applications**	1.12 (0.89 to 1.41) p=0.34	1.12 (0.94 to 1.35) p=0.21
**Writing**	1.10 (0.87 to 1.41) p=0.42	1.00 (0.84 to 1.19) p=0.99
**'Total'**	1.26 (1.03 to 1.55) p=0.03	1.19 (1.03 to 1.37) p=0.02
**UCAT**		
**Abstract Reasoning**	1.05 (0.86 to 1.29) p=0.63	1.10 (0.97 to 1.24) p=0.13
**Decision Analysis**	1.06 (0.83 to 1.34) p=0.66	1.14 (>1.00 to 1.30) p=0.05
**Quantitative Reasoning**	1.15 (0.91 to 1.46) p=0.25	1.07 (0.94 to 1.23) p=0.31
**Verbal Reasoning**	1.34 (1.04 to 1.71) p=0.02	1.15 (>1.00 to 1.32) p=0.05
**Total**	1.25 (0.96 to 1.64) p=0.10	1.22 (1.05 to 1.42) p=0.01
**'Rebalanced' total**	1.34 (1.03 to 1.75) p=0.03	1.22 (1.05 to 1.41) p=0.01

Table B3. Comparison of odds ratios on non-imputed and imputed data, predicting passing *MRCP: PACES* at the first attempt. All models are adjusted for prior educational attainment.

For peer review only

C: Results from 'score relative to pass'

Tables C1 – C3 display results from linear regression models predicting score relative to pass at each section of the MRCP(UK) at the first attempt. Regression coefficients are shown unadjusted and adjusted for prior educational attainment.

Score relative to pass	MRCP: Part 1	
	Unadjusted β	β adjusted for PEA
BMAT		
Aptitude and Skills	1.25 (0.68 to 1.82) p<0.001	1.07 (0.49 to 1.65) p<0.001
Scientific Knowledge and Applications	2.02 (1.43 to 2.60) p<0.001	1.83 (1.24 to 2.42) p<0.001
Writing	-0.04 (-0.60 to 0.51) p=0.88	-0.06 (-0.62 to 0.49) p=0.83
'Total'	1.95 (1.40 to 2.50) p<0.001	1.76 (1.20 to 2.32) p<0.001
UCAT		
Abstract Reasoning	0.25 (-0.25 to 0.75) p=0.38	0.14 (-0.36 to 0.64) p=0.59
Decision Analysis	0.46 (-0.10 to 1.03) p=0.11	0.37 (-0.19 to 0.94) p=0.19
Quantitative Reasoning	1.62 (1.03 to 2.21) p<0.001	1.51 (0.92 to 2.10) p<0.001
Verbal Reasoning	1.03 (0.43 to 1.62) p<0.001	0.91 (0.31 to 1.50) p<0.01
Total	1.46 (0.80 to 2.11) p<0.001	1.27 (0.61 to 1.93) p<0.001
'Rebalanced' total	1.47 (0.82 to 2.12) p<0.001	1.30 (0.64 to 1.95) p<0.001

Table C1. Results from multilevel linear regression models predicting score relative to pass on *MRCP: Part 1* at the first attempt, unadjusted and adjusted for prior educational attainment (PEA).

For peer review only

Score relative to pass	MRCP: Part 2	
	Unadjusted β	β adjusted for PEA
**BMAT**		
**Aptitude and Skills**	1.80 (1.27 to 2.32) p<0.001	1.75 (1.21 to 2.28) p<0.001
**Scientific Knowledge and Applications**	1.71 (1.16 to 2.26) p<0.001	1.71 (1.16 to 2.27) p<0.001
**Writing**	0.30 (-0.26 to 0.87) p=0.29	0.38 (-0.19 to 0.95) p=0.19
**'Total'**	1.90 (1.42 to 2.38) p<0.001	1.87 (1.37 to 2.36) p<0.001
**UCAT**		
**Abstract Reasoning**	0.05 (-0.45 to 0.55) p=0.84	0.04 (-0.47 to 0.55) p=0.87
**Decision Analysis**	0.37 (-0.21 to 0.95) p=0.21	0.38 (-0.20 to 0.97) p=0.20
**Quantitative Reasoning**	1.14 (0.53 to 1.74) p<0.001	1.17 (0.56 to 1.78) p<0.001
**Verbal Reasoning**	1.66 (1.04 to 2.27) p<0.001	1.62 (1.00 to 2.25) p<0.001
**Total**	1.22 (0.56 to 1.88) p<0.001	1.25 (0.58 to 1.92) p<0.001
**'Rebalanced' total**	1.60 (0.95 to 2.26) p<0.001	1.69 (1.03 to 2.36) p<0.001

Table C2. Results from multilevel linear regression models predicting score relative to pass on *MRCP: Part 2* at the first attempt, unadjusted and adjusted for prior educational attainment (PEA).

For peer review only

Score relative to pass	MRCP: PACES	
	Unadjusted β	β adjusted for PEA
**BMAT**		
**Aptitude and Skills**	2.37 (0.88 to 2.89) p<0.01	2.36 (0.85 to 3.87) p<0.01
**Scientific Knowledge and Applications**	1.68 (0.11 to 3.36) p=0.04	1.66 (0.07 to 3.26) p=0.04
**Writing**	1.22 (-0.47 to 2.92) p=0.16	1.17 (-0.54 to 2.88) p=0.18
**'Total'**	2.19 (0.80 to 3.57) p<0.01	2.19 (0.78 to 3.60) p<0.01
**UCAT**		
**Abstract Reasoning**	0.68 (-0.77 to 2.14) p=0.36	0.62 (-0.85 to 2.10) p=0.41
**Decision Analysis**	0.86 (-0.84 to 2.55) p=0.32	0.77 (-0.96 to 2.50) p=0.38
**Quantitative Reasoning**	0.79 (-0.91 to 2.49) p=0.36	0.74 (-0.97 to 2.44) p=0.40
**Verbal Reasoning**	3.00 (1.33 to 4.67) p<0.001	2.99 (1.28 to 4.70) p<0.001
**Total**	2.18 (0.36 to 4.00) p=0.02	2.14 (0.26 to 4.02) p=0.03
**'Rebalanced' total**	2.88 (1.09 to 4.66) p<0.01	2.89 (1.04 to 4.74) p<0.01

Table C3. Results from multilevel linear regression models predicting score relative to pass on *MRCP: PACES* at the first attempt, unadjusted and adjusted for prior educational attainment (PEA).

For peer review only

STROBE 2007 (v4) Statement—Checklist of items that should be included in reports of *cohort studies*

Section/Topic	Item #	Recommendation	Reported on page #
**Title and abstract**	1	(*a*) Indicate the study's design with a commonly used term in the title or the abstract	1
		(*b*) Provide in the abstract an informative and balanced summary of what was done and what was found	2-3
**Introduction**			
Background/rationale	2	Explain the scientific background and rationale for the investigation being reported	4-9
Objectives	3	State specific objectives, including any prespecified hypotheses	9
**Methods**			
Study design	4	Present key elements of study design early in the paper	10, Fig 1
Setting	5	Describe the setting, locations, and relevant dates, including periods of recruitment, exposure, follow-up, and data collection	10, Fig 1
Participants	6	(*a*) Give the eligibility criteria, and the sources and methods of selection of participants. Describe methods of follow-up	10, Fig 1
		(*b*) For matched studies, give matching criteria and number of exposed and unexposed	n/a
Variables	7	Clearly define all outcomes, exposures, predictors, potential confounders, and effect modifiers. Give diagnostic criteria, if applicable	10-12
Data sources/ measurement	8*	For each variable of interest, give sources of data and details of methods of assessment (measurement). Describe comparability of assessment methods if there is more than one group	10-12
Bias	9	Describe any efforts to address potential sources of bias	11-13
Study size	10	Explain how the study size was arrived at	12-13, Fig 1
Quantitative variables	11	Explain how quantitative variables were handled in the analyses. If applicable, describe which groupings were chosen and why	10-13
Statistical methods	12	(*a*) Describe all statistical methods, including those used to control for confounding	12-13
		(*b*) Describe any methods used to examine subgroups and interactions	12-13
		(*c*) Explain how missing data were addressed	13
		(*d*) If applicable, explain how loss to follow-up was addressed	n/a
		(*e*) Describe any sensitivity analyses	13
**Results**			

For peer review only

Participants	13*	(a) Report numbers of individuals at each stage of study—eg numbers potentially eligible, examined for eligibility, confirmed eligible, included in the study, completing follow-up, and analysed	Fig 1
		(b) Give reasons for non-participation at each stage	n/a
		(c) Consider use of a flow diagram	Fig 1
Descriptive data	14*	(a) Give characteristics of study participants (eg demographic, clinical, social) and information on exposures and potential confounders	Table 1
		(b) Indicate number of participants with missing data for each variable of interest	Table 1
		(c) Summarise follow-up time (eg, average and total amount)	n/a
Outcome data	15*	Report numbers of outcome events or summary measures over time	13
Main results	16	(*a*) Give unadjusted estimates and, if applicable, confounder-adjusted estimates and their precision (eg, 95% confidence interval). Make clear which confounders were adjusted for and why they were included	Fig 2, 3, Supplementary appendix
		(*b*) Report category boundaries when continuous variables were categorized	n/a
		(*c*) If relevant, consider translating estimates of relative risk into absolute risk for a meaningful time period	n/a
Other analyses	17	Report other analyses done—eg analyses of subgroups and interactions, and sensitivity analyses	16-17, supplementary appendix
**Discussion**			
Key results	18	Summarise key results with reference to study objectives	17-18
**Limitations**			
Interpretation	20	Give a cautious overall interpretation of results considering objectives, limitations, multiplicity of analyses, results from similar studies, and other relevant evidence	17-22
Generalisability	21	Discuss the generalisability (external validity) of the study results	22-24
**Other information**			
Funding	22	Give the source of funding and the role of the funders for the present study and, if applicable, for the original study on which the present article is based	31-32

*Give information separately for cases and controls in case-control studies and, if applicable, for exposed and unexposed groups in cohort and cross-sectional studies.

For peer review only

Note: An Explanation and Elaboration article discusses each checklist item and gives methodological background and published examples of transparent reporting. The STROBE checklist is best used in conjunction with this article (freely available on the Web sites of PLoS Medicine at http://www.plosmedicine.org/, Annals of Internal Medicine at http://www.annals.org/, and Epidemiology at http://www.epidem.com/). Information on the STROBE Initiative is available at www.strobe-statement.org.

For peer review only

BMJ Open

Can achievement at medical admissions tests predict future performance in postgraduate clinical assessments? A UK-based national cohort study

Journal:	*BMJ Open*
Manuscript ID	bmjopen-2021-056129.R1
Article Type:	Original research
Date Submitted by the Author:	30-Nov-2021
Complete List of Authors:	Paton, Lewis; University of York, Health Sciences<br>McManus, Ian; University College London, Psychology<br>Cheung, Kevin; University of Cambridge, Cambridge Assessment<br>Smith, Daniel; General Medical Council, Education and Standards<br>Tiffin, Paul; University of York, Health Sciences
**Primary Subject Heading**:	Medical education and training
Secondary Subject Heading:	Medical education and training
Keywords:	MEDICAL EDUCATION & TRAINING, GENERAL MEDICINE (see Internal Medicine), EDUCATION & TRAINING (see Medical Education & Training)

SCHOLARONE™
Manuscripts

I, the Submitting Author has the right to grant and does grant on behalf of all authors of the Work (as defined in the below author licence), an exclusive licence and/or a non-exclusive licence for contributions from authors who are: i) UK Crown employees; ii) where BMJ has agreed a CC-BY licence shall apply, and/or iii) in accordance with the terms applicable for US Federal Government officers or employees acting as part of their official duties; on a worldwide, perpetual, irrevocable, royalty-free basis to BMJ Publishing Group Ltd ("BMJ") its licensees and where the relevant Journal is co-owned by BMJ to the co-owners of the Journal, to publish the Work in this journal and any other BMJ products and to exploit all rights, as set out in our licence.

The Submitting Author accepts and understands that any supply made under these terms is made by BMJ to the Submitting Author unless you are acting as an employee on behalf of your employer or a postgraduate student of an affiliated institution which is paying any applicable article publishing charge ("APC") for Open Access articles. Where the Submitting Author wishes to make the Work available on an Open Access basis (and intends to pay the relevant APC), the terms of reuse of such Open Access shall be governed by a Creative Commons licence – details of these licences and which Creative Commons licence will apply to this Work are set out in our licence referred to above.

Other than as permitted in any relevant BMJ Author's Self Archiving Policies, I confirm this Work has not been accepted for publication elsewhere, is not being considered for publication elsewhere and does not duplicate material already published. I confirm all authors consent to publication of this Work and authorise the granting of this licence.

For peer review only

Can achievement at medical admissions tests predict future performance in postgraduate clinical assessments? A UK-based national cohort study

Lewis W Paton, I C McManus, Kevin Y F Cheung, Daniel T Smith, Paul A Tiffin

LWP (corresponding author): Department of Health Sciences, University of York, UK, ORCID: https://orcid.org/0000-0002-3328-5634, lewis.paton@york.ac.uk

ICM: Research Department of Medical Education, University College London, UK. ORCID: https://orcid.org/0000-0003-3510-4814

KYFC: Cambridge Assessment English, University of Cambridge, UK. ORCID: https://orcid.org/0000-0002-9548-2932

DTS: General Medical Council, UK. ORCID: https://orcid.org/0000-0003-1215-5811

PAT: Department of Health Sciences, University of York and Health Professions Education Unit, Hull York Medical School, UK. ORCID: https://orcid.org/0000-0003-1770-5034

For peer review only

ABSTRACT

Objective To determine if scores on two undergraduate admissions tests (BioMedical Admissions Test, BMAT and University Clinical Aptitude Test, UCAT) predict performance on the postgraduate Membership of the Royal College of Physicians (MRCP) examination, including the clinical examination Practical Assessment of Clinical Examination Skills (PACES).

Design National cohort study.

Setting Doctors who graduated medical school between 2006 and 2018.

Participants 3,045 doctors who had sat BMAT, UCAT and the MRCP.

Primary outcome measures Passing each section of the MRCP at the first attempt, including the clinical assessment PACES.

Results Several BMAT and UCAT subtest scores displayed incremental predictive validity for performance on the first two (written) parts of the MRCP. Only *aptitude and skills* on BMAT (OR 1.34, 1.08-1.67, p=0.01) and *verbal reasoning* on UCAT (OR 1.34, 1.04-1.71, p=0.02) incrementally predicted passing PACES at the first attempt.

Conclusions Our results imply that the abilities assessed by *aptitude and skills* and *verbal reasoning* may be the most important cognitive attributes, of those routinely assessed at selection, for predicting future clinical performance. Selectors may wish to consider placing particular weight on scales assessing these attributes if they wish to select applicants likely to become more competent clinicians. These results are potentially relevant in an international context too, since many admissions tests used globally, such as the Medical College Admission Test, assess similar abilities.

For peer review only

Strengths and limitations of this study

- We had access to a national sample of medical graduates.
- Restricting our analyses to those who had sat both BMAT and UCAT allowed for a comparison of results across the two admissions tests.
- While our results are likely to be generalisable to all applicants who sit BMAT, it is unclear if this is the case for all applicants who sit UCAT.
- While a substantial proportion of doctors sit the MRCP as part of their training, the generalisability of our results to other Royal College examinations is unclear.

For peer review only

Traditionally, access to medical school has been based on educational attainment in secondary (high) school. Academic entry standards have partly been driven by strong competition for places, as well as the intellectual demands of the courses. Moreover, prior educational attainment has been shown to predict performance in undergraduate and postgraduate medical training [1-6]. Applicants to medical school are also required to sit additional standardised tests as part of the selection process. These have been introduced to help selectors further differentiate between educationally high performing candidates and choose those considered to have the aptitude for a medical career. At times it has also been hoped that such tests will facilitate widening access to medicine, if they are less sensitive to certain sociodemographic characteristics, compared to traditional metrics of attainment [7].

The two main selection assessments currently used for entry into undergraduate medicine in UK medical schools are BioMedical Admissions Test (BMAT) [8] and University Clinical Aptitude Test (UCAT – previously known as UKCAT) [9]. Both tests consist of a number of subtests [10 11], described in Table 1. These admissions tests are used in conjunction with other selection measures, such as interviews (including multiple mini-interviews), personal statements, references and educational attainment. Applicants to undergraduate medicine in the UK may apply to up to four different universities in a particular application cycle. As such, candidates may sit one or both of BMAT and UCAT, depending on the entry requirements of their chosen medical schools. These entry requirements may change year on year. Successful applicants to medical school then embark on an undergraduate medical course generally consisting of five years. Medical graduates then undertake the two-year Foundation Programme, before embarking on specialty training in their chosen field of medicine. Each specialty is aligned with a particular Royal College, and as part of their specialty training, doctors must pass the Royal College membership examinations. Some of

For peer review only - http://bmjopen.bmj.com/site/about/guidelines.xhtml

these exams can be taken during the Foundation Programme prior to commencing specialty training.

For any test used in admissions processes, it is important that scores from the test predict outcomes of interest. It is reasonably well established internationally that admission tests predict undergraduate medical education outcomes. In the case of BMAT, it has previously been reported that scores are predictive of first year examination scores [12], and that scores on *scientific knowledge and applications* correlate with examination marks more than scores on *aptitude and skills.* UCAT scores are predictive of undergraduate performance throughout undergraduate medical school [13-15]. In the case of the UCAT, the ability of the scores have been shown to demonstrate incremental validity, above and beyond that provided by prior educational attainment, and additionally that scores on the *verbal reasoning* subtest had the strongest relationship with undergraduate academic achievement[13]. However, as medical training extends beyond the initial undergraduate experience, it is important that we consider the predictive validity of admissions tests for relevant postgraduate outcomes. In this regard, there is relatively little published evidence. There are some indications that BMAT and UCAT scores are associated with performance in the written components of two UK Royal College membership examinations (equivalent to US board examination) [16 17]. Thus, there is some emerging evidence that these assessments add value to the selection process when determining which applicants may be best suited to the academic challenges of early medical training. However, so that selectors have as much information as possible, it is important that further research into the predictive validity of BMAT and UCAT for postgraduate outcomes is carried out.

Furthermore, performance in written examinations is perhaps less important than how an individual behaves in actual clinical practise. Whilst relevant semantic knowledge is vital in order to deliver safe and effective care, other skills, such as problem-solving ability, are also

For peer review only - http://bmjopen.bmj.com/site/about/guidelines.xhtml

likely to be crucial. Potential doctors are increasingly assessed on these abilities in a variety of ways, notably by selection assessments. Indeed, the admissions tests currently most widely used consist of a number of subsections, some of which aim to evaluate more fluid concepts of cognitive ability, in addition to sections evaluating the ability to recall factual knowledge.

However, linking performance on admissions assessments to aspects of actual clinical care and patient outcomes is extremely challenging for several reasons. One major barrier would be distinguishing between the impact of the individual doctor, versus the team or service, on a care outcome. Nevertheless, simulation-based examinations may serve as a plausible proxy for actual clinical behaviour, albeit reflecting 'maximal' rather than 'typical' performance. Such examinations are commonly taken as part of postgraduate medical training. One of the most widely taken Royal College examinations in the UK is the *Membership of the Royal Colleges of Physicians of the UK (MRCP(UK))* [18]. The MRCP(UK), referred to hereafter as the MRCP, consists of three parts [19], including the *Practical Assessment of Clinical Examination Skills (PACES)* (see Table 1). Trainees must complete all parts of the examination to advance to higher specialty training in the UK.

[INSERT TABLE 1 HERE]

The creation of the UK Medical Education Database (UKMED) [20], has generated a longitudinal linked dataset relating to individuals from application to medical school, through graduation and into post qualification medical training. The UKMED has now matured to the point that there is a valuable opportunity to evaluate the extent to which performance at selection into medical school is reflected in performance in clinical assessments, many years later, following graduation. Specifically, it would be possible to evaluate which components

For peer review only - http://bmjopen.bmj.com/site/about/guidelines.xhtml

of selection assessments have the most incremental predictive validity. In doing so, we draw from the 'individual differences' field, and in particular the psychometrics of intelligence, and how these can be applied to the interpretation of cognitive test scores, such as those assessed by BMAT and UCAT [21]. That is, what does the observed performance on different aspects of such assessments tell us about the likely underlying traits and abilities of the test-taker, relative to others?

This study thus had the following specific aims:

- To assess to what extent performance on the different components of BMAT and UCAT predict performance on the MRCP examinations, with a particular focus on the clinical assessment PACES.
- To determine if this predictive validity was incremental, over that provided by secondary (high) school educational attainment- the other main intellectual ability measure commonly used in the selection of medical students.

The findings would have clear implications for how scores from such tools are used within the selection process, and in particular, the relative weight that should be placed on those components mainly testing semantic recall, compared to problem-solving skills. In the context of UK medical selection, understanding the predictive profiles of BMAT and UCAT would help selectors choose an admission test they felt best suited their local requirements. However, as similar cognitive assessments are used globally in medical selection our results would have international relevance.

METHODS

Note that all data and results presented in this paper are blunted in line with UKMED statistical disclosure controls [22].

For peer review only - http://bmjopen.bmj.com/site/about/guidelines.xhtml

Data availability and preparation

All data used in the analyses were obtained from the UK Medical Education Database (UKMED) [20]. Data were available for 69, 885 medical graduates between 2006 and 2018. Of these, 13,090 graduates had MRCP outcome data. We restricted analyses to those students who had sat both BMAT and UCAT; that is 3,045 graduates. This was to enable a comparison of the predictive validity between the two admissions tests. Figure 1 shows the flow of data through this study. It should be noted that the majority of graduates who had sat BMAT had also sat the UCAT, though the reverse was not true.

[INSERT FIGURE 1 HERE]

Outcome variables

The primary outcomes of interest were the odds of passing each section of the MRCP at the first attempt. Performance at the first attempt at each section of the MRCP was chosen as this has been shown to be a good indicator of overall performance [23]. Predictions relating to a secondary outcome, the continuous 'score relative to pass at first sitting', for the exam components were also analysed. The results relating to these latter, continuous outcomes are presented in Supplemental Digital Appendix 1.

Predictor variables

In order to make our results relevant to selectors, performance at the most recent attempt at each section of both admissions tests was used, since it is these scores that are associated with admission to medical school. The subtest scores of BMAT (*aptitude and skills*, *scientific knowledge and applications* and *writing*) and the UCAT (*abstract reasoning*, *decision*

For peer review only - http://bmjopen.bmj.com/site/about/guidelines.xhtml

analysis, *quantitative reasoning* and *verbal reasoning*) were standardised as z-scores (mean 0, standard deviation 1) within each cohort of test takers (including unsuccessful applicants) and used as independent variables in our model. This standardisation process was intended to increase the comparability of selection assessment scores over time, as these are known to change.

As medical school selectors often base decisions on combined scores on the admissions tests, we also included a number of summed scales. We included summed BMAT *aptitude and skills and scientific knowledge and applications* score, denoted 'total BMAT score'. We also included *total UCAT score* on all four scales. Additionally, it has been previously been shown that UCAT can be conceptualised as measuring two dimensions of cognitive functioning; verbal and non-verbal reasoning [24]. Thus, we also created a 'rebalanced' total UCAT score in order to adjust for the high relative weighting on non-verbal reasoning created by simply summing the scores from all four subtests. This 'rebalanced' score is the average of the three non-verbal scales (*abstract reasoning, decision analysis, quantitative reasoning*) added to the *verbal reasoning* score. All these summary scores were standardised within each cohort.

It was important to assess the *incremental* predictive validity of the two admissions tests, over and above that provided by conventional education achievement at secondary school. Therefore, two measures of prior academic attainment were used as covariates in the predictive models evaluated. Firstly, A-level performance was used for those students from English secondary schools. As in previous research [14], we used each entrant's best three A-level results, excluding 'General Studies'. We restricted analyses to those with A-levels sat prior to the introduction of A* grades in 2010. Achieved grades were awarded a tariff score (A=10, B=8 etc.). Thus, the maximum tariff for each entrant was 30. The tariff scores were then standardised as z-scores by cohort of applicants. The second measure of prior academic attainment we used was General Certificate of Secondary Education (GCSE) performance.

For peer review only - http://bmjopen.bmj.com/site/about/guidelines.xhtml

Data were available on the sum of the nine best GCSE grades each candidate scored, where A*=6, A=5, B=4, and so on. ‘Double awards’ were counted as two separate GCSEs. Summed performance was standardised as z-scores by cohort of applicants. The dates of sitting of GCSEs were not available in the data. Thus, we determined cohorts by year of first sitting of an admissions test, assuming that the majority of candidates from the same GCSE cohort will belong to the same admissions test cohort. This approach would not achieve perfect accuracy, since some individuals will choose to take a gap year or defer entry into university. However, it represented the optimum way, pragmatically to estimate the year of GCSE sitting, in order to attempt to adjust for ‘grade inflation’.

Data analysis

Multilevel logistic regression models were built for the dichotomous outcome ‘pass at first sitting’, for each section of the MRCP. Multilevel models were used to allow the effects of the predictors to vary across medical school attended (via a random intercept), as previous research has shown that average MRCP scores are, to some extent, related to the particular medical school attended [25].

Models were built for each subtest score of BMAT and the UCAT, as well as the summary measures of the overall scores described above. Models were built that were both unadjusted and adjusted for prior educational attainment (GCSE and A-level performance). Only information from graduates with complete prior educational data were included in the models, to ensure full nesting of the models. Therefore, n=1,320 individuals were included in our final analysis, including 1,315 who had data for Part 1 of the MRCP, 825 who had Part 2 data, and 460 who had PACES data. Of these, at the first attempt, 975 passed Part 1, 715 passed Part 2, and 310 passed PACES.

Missing data

For peer review only - http://bmjopen.bmj.com/site/about/guidelines.xhtml

Multiple imputation was used as a form of sensitivity analysis to assess the impact of missing prior educational data (GCSEs and A-levels) on our results. Standardised GCSE and A-level scores were imputed using multiple imputations by chained equations with linear regression from other educational data and sociodemographic variables. The number of imputations was chosen to ensure stability of results. We analysed the imputed dataset in the manner described above, and compared regression coefficients with the models performed on non-imputed data. This allowed us to assess the impact of missing educational data on our results.

All analyses were performed in the Health Informatics Centre Safe Haven using Stata version 14 [26].

Patient and Public Involvement

None.

RESULTS

Descriptive statistics

[INSERT TABLE 2 HERE]

Table 2 displays descriptive statistics for our cohort. Compared to the wider cohort of medical graduates, those who sat both BMAT and UCAT had higher academic attainment. These differences were all statistically significant at the p=0.05 level on Mann-Whitney U testing. Of those graduates who had sat both BMAT and UCAT, those who had also sat the MRCP had somewhat higher scores on the first two sections of BMAT, but lower scores on

For peer review only - http://bmjopen.bmj.com/site/about/guidelines.xhtml

For peer review only

the writing component of BMAT as well as each section of the UCAT. Again, these differences were statistically significant at the p=0.05 level on formal testing.

Predictive validity of BMAT and UCAT for MRCP performance

Figure 2 displays the predictive and incremental validity for BMAT subtest and summary scores for passing each section of the MRCP at the first attempt. Figure 3 displays the same values for the UCAT subtest and summary scores. In both figures, the circle represents the odds ratio from the univariable (or unadjusted) multilevel logistic regression models. The triangle represents the odds ratio from the multivariable (or adjusted) models. Tables displaying these results are available in Supplemental Digital Appendix 1.

[INSERT FIGURE 2 HERE]

As can be seen in Figure 2, passing the first part of the MRCP is incrementally predicted by scores on *aptitude and skills* (OR 1.22, 1.04 to 1.44, p=0.02) and scores on *scientific knowledge and applications* (OR 1.49, 1.25 to 1.78, p<0.001). This later result can be interpreted as follows. For every standard deviation above the mean an individual scored on *scientific knowledge and applications,* their odds of passing Part 1 of the MRCP at the first attempt increased by around 49%. Similarly, performance on both *aptitude and skills* (OR 1.36, 1.05 to 1.75, p=0.02*)* and *scientific knowledge and applications* (OR 1.35, 1.04 to 1.75, p=0.02*)* incrementally predicts passing Part 2 of the MRCP at the first attempt. When it comes to passing the clinical assessment PACES, evidence of predictive validity was only observed for the *aptitude and skills* score (OR 1.34, 1.08 to 1.67, p=0.01), and this result is independent of prior educational attainment. 'Total' BMAT score (that is, total score across the first two sections) is a significant predictor of passing all sections of the MRCP at the first

For peer review only - http://bmjopen.bmj.com/site/about/guidelines.xhtml

attempt. In contrast, performance on the writing component of BMAT is not a significant predictor for any of the outcome variables analysed.

[INSERT FIGURE 3 HERE]

In the case of UCAT (Figure 3), *quantitative reasoning* (OR 1.37, 1.16 to 1.62, p<0.001) and *verbal reasoning* (OR 1.24, 1.05 to 1.48, p=0.01) scores incrementally predicted passing the first, knowledge-based, part of the MRCP at the first attempt. Higher v*erbal reasoning* scores (OR 1.55, 1.17 to 2.04, p<0.01) are associated with a higher odds of passing Part 2 (applied knowledge) of the MRCP at the first attempt. However, a higher score on the UCAT *abstract reasoning* subtest was associated with *lower* odds of passing Part 2 of the MRCP examination at the first attempt, independently of prior educational attainment (OR 0.79, 0.64 to 0.99, p=0.04). Only the UCAT *verbal reasoning* scores were statistically significantly associated with the odds of passing the clinical assessment, PACES, at first attempt (OR 1.34, 1.04 to 1.71, p=0.02). In this case, the odds of passing the PACES at first attempt increased by around a third for every standard deviation above the mean achieved on the UCAT *verbal reasoning* subtest, for applicants sitting the test. The total UCAT score is a significant independent predictor of passing part 1 of the MRCP, but not subsequent sections of the exam. However, the 'rebalanced' UCAT score, which weights verbal and non-verbal performance equally, is a statistically significant independent predictor of passing all sections of the MRCP, including PACES (OR 1.34, 1.03 to 1.75, p=0.03). Indeed, the odds of passing the PACES at first attempt increase by around a third for every standard deviation above the mean achieved for applicants sitting the test. No statistically significant associations with the outcomes of interest and the UCAT *decision analysis* scores were observed.

For peer review only - http://bmjopen.bmj.com/site/about/guidelines.xhtml

Imputation of prior educational attainment

In those who sat both BMAT and UCAT, missing values of A-levels and GCSE scores were imputed. We used chained imputation methods, building models on available sociodemographic variables and educational variables, where present. Imputation results stabilised after 20 iterations. Logistic regression models, controlling for the influence of A-levels and GCSEs, were fitted to the imputed data set to assess the incremental predictive validity of each scale score for predicting whether an individual passed each section of the MRCP on first attempt. As a form of sensitivity analysis, regression coefficients were compared to coefficients obtained when fitting the same model to the non-imputed data (as presented in Figures 2 and 3). Full results from models on imputed data are available in Supplemental Digital Appendix 1.

In general, the results from models fitted to imputed data are broadly similar to those fitted to non-imputed data. Only small differences in effect size or p-values were observed when predicting performance on Part 1 of the MRCP. No overall changes in statistical significance were observed. For Part 2 of the MRCP, no differences were observed in the predictive validity of BMAT subtest scores. There are some differences in how the UCAT subtest scores behave, however. The a*bstract reasoning* subtest score becomes a non-statistically significant predictor, and the *quantitative reasoning* and total UCAT become statistically significant predictors ($p<0.05$). For predicting the performance at PACES, the UCAT *decision analysis* score becomes a predictor of only borderline statistical significance, and the total UCAT score becomes statistically significant. Again, negligible differences were observed in relation to the predictive validity of BMAT subtest scores.

DISCUSSION

For peer review only - http://bmjopen.bmj.com/site/about/guidelines.xhtml

In this study, we observed that both BMAT and UCAT scores have incremental predictive validity for performance on the MRCP. As might be expected, scores on different sections of the admissions tests predict performance on the various sections of the MRCP. Performance on the BMAT *aptitude and skills* subtest and on the UCAT *verbal reasoning* subtest predicted the odds of passing all sections of the MRCP at first attempt, independently of prior educational attainment.

Of particular interest was the relationship between the selection assessment scores and performance on the PACES, as an assessment of practical clinical skills. In those graduates who had originally sat both BMAT and the UCAT, we observed that scores on BMAT *aptitude and skills* (OR 1.34, 1.08 to 1.67) and the UCAT *verbal reasoning* (OR 1.34, 1.04 to 1.71) subtests were incrementally predictive of passing the PACES at the first attempt. We also noted that the summed scores, across the first two BMAT subtests, predicted performance on all sections of the MRCP. It was also observed that a 'rebalanced' UCAT summary score, which addresses the substantial weight on non-verbal reasoning, appears to add value over using the usual summary, total UCAT score. This was especially evident when predicting performance on the PACES, where only the 'rebalanced' total was incrementally predictive.

Consistently, BMAT *aptitude and skills* and UCAT *verbal reasoning* subtest scores showed predictive validity when considering all the MRCP related outcomes of interest. This is in line with findings from a previous study of the predictive validity of the UCAT scores, where performance on the *verbal reasoning* subtest had the strongest relationship with academic achievement throughout medical school study [13]. As with the present findings, this predictive ability was incremental over and above that provided by prior educational attainment at secondary (high) school. Thus, it is reasonable to infer that these assessment sections evaluate abilities that are both relevant to future performance in clinical tests of both pure and applied

For peer review only - http://bmjopen.bmj.com/site/about/guidelines.xhtml

semantic knowledge and procedural skills. Moreover, at least to some extent, they must measure traits or abilities not fully covered by the, largely science-based, subjects taken at secondary school, especially at Advanced level. Previous research has shown that the BMAT *aptitude and skills* subtest can be conceptualised as measuring 'thinking skills' [27], that is, problem solving ability. The UCAT *verbal reasoning* subtest, as mentioned earlier, evaluates the ability to make inferences and draw conclusions from written information, and can be considered related, though somewhat distinct from, problem solving that does not heavily rely on verbal comprehension skills [24]. Thus, both selection assessment subtests test cognitive, problem solving skills, rather than semantic, factual recall, though verbal reasoning does not seek to assess such non-verbal skills. Nevertheless, both subtests generated scores with a similar ability to predict subsequent performance in PACES.

Our findings can be considered alongside results from a study assessing the relationship between BMAT and UCAT scores and performance on the Membership of the Royal College of Surgeons (MRCS) examination. [17] In this instance, using data from a comparable time period, the authors observed a statistically significant relationship between admissions test scores and performance on the written component of the MRCS, and evidence of *aptitude and skills* and *abstract reasoning* scores incrementally predicting performance on the clinical examination component.. It is not reported how this study of MRCS performance controlled for the fact that some candidates had sat one of the admissions tests, while others had sat both. Thus, comparisons across the two admissions tests within that study, and indeed direct comparisons with this study, are difficult to make. Nevertheless, taken together, it appears there is some emerging evidence regarding the relationship between admissions test scores and performance on postgraduate clinical assessments more broadly. In both this study and the MRCS study, it is the scores on BMAT *aptitude and skills* that predicts performance at the Royal College clinical assessment, rather than scores on the component of BMAT which

For peer review only - http://bmjopen.bmj.com/site/about/guidelines.xhtml

assesses more factual recall. The UCAT scale scores which possess predictive power for the clinical assessment differs between the MRCP, where *verbal reasoning* scores are statistically significant, and the MRCS, where it is the *abstract reasoning* scores. Such a difference may by explained simply by the different skills required to pass a surgical clinical assessment and those needed for a more general internal medicine clinical assessment. However, taking all the results together, it suggests that it is indeed cognitive, problem solving skills which are particularly important for predicting performance at Royal College clinical assessments, rather than semantic, factual recall.

There are some advanced school qualifications, such as 'thinking skills' [28] that may evaluate such cognitive, problem solving skills, though they are not generally accepted as part of entry requirements by UK medical schools. However, it may be that the high school maths and science exams, generally taken by medical school applicants, do not always test these abilities fully, typically placing more emphasis on testing non-verbal problem-solving skills and semantic factual recall. Also, as previously illustrated via modelling studies, academic or educational performance is not simply a direct result of intellectual (cognitive) ability, but can mediated by separate factors such as secondary school attended [29] or personality traits such as conscientiousness [30 31]. Indeed, more recently there have been attempts to identify the genetic basis of 'non-cognitive' abilities by partitioning education achievement from intellectual ability [32]. Moreover, when predicting performance at 'high fidelity' clinical assessments, abilities related to inter-personal functioning, may be stronger predictors than levels of relevant semantic knowledge [33]. Within the individual differences tradition such traits and abilities are often conceptualised within an 'emotional intelligence' framework [34]. However, the distinction between such 'non-cognitive' and 'cognitive' abilities is not always well demarcated [35]. This is especially interesting in the case of verbal ability. Indeed, the earliest attempts to measure 'social intelligence' found it difficult to delineate between verbal

For peer review only - http://bmjopen.bmj.com/site/about/guidelines.xhtml

For peer review only

ability and experimental measures of interpersonal competence [36]. Individuals are often judged in regards to their ‘social skills’ on the basis of the language they use, as much as their behaviour. Thus, in this case, to some extent, verbal reasoning, though conventionally conceptualised as a ‘cognitive ability’, could still be an indicator of interpersonal skills that would be important in performing well in tests that involved simulated patients or clinical situations.

A counterintuitive result observed was that for a higher UCAT *abstract reasoning* score to be associated with a lower odds of passing MRCP Part 2 at the first attempt. However, once missing prior educational data were imputed the abstract reasoning scores became a non-statistically significant predictor of MRCP Part 2 performance. This suggests that caution should be used when interpreting the former result. Additionally, scores on the UCAT *decision analysis* subtest, as well as UCAT total score, became significant predictors of PACES performance in the imputed dataset.

Adjusting for A-level performance alone had very little impact on the results. This is why only the findings adjusted for both A-level and GCSE performance were presented. This is almost certainly due to the lack of variation in A-level performance for the final sample of doctors included in the analysis. For example, over 70% of medical graduates with A-level data from before 2010 achieved the maximum available grade of AAA. This lack of observed variation would have been further constrained in the final sample of doctors sitting the MRCP relatively early in their careers, who may have been particularly academically well performing. Adjusting for GCSE performance, where the exams had been taken around the age of 16 years, had a somewhat greater impact on the adjusted results, particularly when predicting performance on Part 1 of the MRCP. This increased impact of adjusting for GCSE, rather than A-level performance, is likely due to greater variation in grades across the cohort. For future analyses it should be noted that, from 2010 onwards, the introduction of an A*

For peer review only - http://bmjopen.bmj.com/site/about/guidelines.xhtml

grade at A-levels will have increased the variability of the grades obtained by medical students and doctors.

Strengths and potential limitations

We restricted our analytic sample to those individuals who sat both BMAT and UCAT. This allowed for comparison of results across the two admissions tests. However, by restricting the analysis in this way there is a risk that our results may not generalise to those individuals who sat just one of the admissions tests. As could be seen, the cohort analysed here had generally higher academic attainment than the wider cohort of medical graduates available within the UKMED. In practice, due to the relatively low proportion of medical schools using BMAT, compared to the UCAT in the UK, currently relatively few medical applicants in the UK sit only BMAT, but a much greater number of applicants sit only the UCAT. As such, our results are likely to be generalisable to the wider cohort of all applicants who sit BMAT. However, the extent to which our findings would generalise to all applicants who sit the UCAT is uncertain. A number of non-statistically significant trends were noted between selection assessment scores and performance on membership exam components. Due to the stage of maturity of the cohorts being followed via the UKMED the numbers of individuals having sat the MRCP at least once were relatively low. This may mean the study was underpowered to detect relatively small effect sizes. Nevertheless, it is doubtful whether such modest effects would be of 'educational significance', even if they were found to be statistically significant. However, our analyses could be replicated at a later stage when more data are available relating to postgraduate clinical examination performance with the UKMED.

The generalisability of these results to other Royal College examinations is also unclear. Although a substantial proportion of doctors sit the MRCP as part of medical training they

For peer review only - http://bmjopen.bmj.com/site/about/guidelines.xhtml

may not be entirely representative of all those in postgraduate training. However, broadly similar results have been observed in relation to performance on the MRCS examination [17] as observed in this study, although, as discussed earlier, caution should be exercised in making direct comparisons between the studies. As more data become available via the UKMED the presence of similar relationships between selection test scores and other Royal College examinations could be evaluated for, and as UKMED continues to mature, a sample including those who sat the MRCP relatively later in their training could additionally be evaluated.

The selection assessment scores were standardised using cohort means and standard deviations. This was the most appropriate method of including scores in analyses across years. However, some limitations of this approach should be acknowledged. The approach assumes that the ability of the population of test takers (on any test construct) is stable year on year. This may not always be the case, partly as the schools which required each test for admission has changed over time. However, such an adjustment was necessary as non-standardised scores would be rendered even less comparable across cohorts. For example, secular trends may be at work. For example, as selection tests have become widely used and established, applicants may have spent more time developing the skills tested by these exams, so later cohorts are likely to have higher ability, on average, compared to previous ones. Our approach to standardisation may have helped address such temporal influences.

Lastly, the rationale for our study mainly rests on the premise that postgraduate clinical assessments may be a proxy for real world performance. Ideally admissions scores would be linked to metrics of actual clinical performance. However, this is extremely challenging in practise. It may be considered that competent performance in a clinical assessment is perhaps a necessary, though not sufficient, condition for actual workplace practise. In this regard it has been reported that in non-UK medical graduates, increased performance in the practical

For peer review only - http://bmjopen.bmj.com/site/about/guidelines.xhtml

component of a licensing examination was protective of the risk of future fitness to practise events [37].

Potential policy implications

When considering evidence for the validity of an assessment it is sometimes useful to apply Kane's validity framework [38]. This is an argument-based approach, whereby a critique of an assessment is placed in the context of the claims made for the test, as well as how the resulting test scores are used in practice. In this regard the claims made for the UCAT are more ambitious than those of the developers of BMAT. Specifically the UCAT developers claim that the assessment is *'seeking to identify the characteristics in applicants which will make the good clinicians and thus to improve the quality of those who enter the professions with the ultimate aim of improving patient care.'* [39]. Our findings here suggest that the UCAT could potentially be useful in identifying medical applicants likely to become good future clinicians, if we accept the assumption that MRCP performances, and in particular performance on PACES, are a reasonable proxy for actual clinical behaviour. Testing this assumption is, as mentioned earlier, non-trivial. However, there is evidence that clinical examinations have face validity with clinicians [40], which suggests this assumption may indeed be plausible.

Nevertheless, the potential of the UCAT to improve medical selection with regards to selecting those applicants likely to make good doctors will not be fully realised unless the resultant scores are reported and used by selectors in the optimally effective way. In particular it should be noted that reweighting the summary UCAT scores into a 'rebalanced' UCAT summary score, which did not overemphasise non-verbal reasoning, appeared to provide greater effect sizes, particularly when predicting performance on Part 2 and the PACES component of the MRCP. Selectors themselves may also wish to consider placing

For peer review only - http://bmjopen.bmj.com/site/about/guidelines.xhtml

more weight on the verbal reasoning component of the UCAT if they wish to realise additional incremental predictive ability for both undergraduate and postgraduate educational performance. However, some caution should be exercised with this approach; previously it was reported that verbal reasoning was the UCAT cognitive subtest score that was most sensitive to socioeconomic status in medical school applicants [7].

In contrast to the UCAT, BMAT claims, less ambitiously, that the assessment is intended to identify those with "*…potential to succeed on medical and health-related courses*." [8]. In this sense, the present findings largely support this claim, though performance on the writing task section seemed to have little relationship with postgraduate performance in this sample. Moreover, despite the modest claimed aspirations, BMAT component scores and summed scores did seem to have some incremental predictive ability regarding future PACES performance. Unsurprisingly, BMAT scientific knowledge and applications section scores tended to predict performance on Part 1 and 2 of the MRCP, which depends on the ability to recall factual information. However, these predictions remained fairly substantial even after correcting for the influence of prior educational attainment. This suggests that such components of selection assessments may add value above secondary school qualifications, probably by providing a more fine-grained metric, which is able to differentiate test takers at the top end of ability. This is in contrast to the UK based school exams, such as A-levels and GCSEs, where many medical school applicants often achieve straight As (or, from 2010 onwards, A*s). Moreover, such semantic knowledge test may be of practical use in selection when considering applications from overseas candidates, where the equivalence of educational qualifications in the sciences may be in uncertain, or difficult to equate. Nevertheless, consideration should be given to the potential impact on the diversity and widening access agenda if knowledge tests are to be included in the selection process. Such tests are likely to be more sensitive to sociodemographic background factors and coaching

For peer review only - http://bmjopen.bmj.com/site/about/guidelines.xhtml

effects compared to measures of problem-solving ability. However, it should be noted that, assessments of the latter are also prone, to some extent, to practise and coaching effects [41]. Moreover, if the overall goal of medical schools is to produce clinically competent doctors, rather than students and trainees who merely do well at exams requiring factual recall, then more emphasis on the scores from assessments evaluating problem solving ability may be advisable.

It is important to note that in this study, we consider only how the cognitive aspects of intelligence, as measured by BMAT and UCAT, relate to a proxy for clinical competence. However, producing clinically competent doctors is a complex multifaceted process. Prospective doctors are increasingly also assessed on 'non-cognitive' skills such as attitudes and knowledge of professionalism [42]. In order to select those most likely to become competent doctors, it is likely that selectors should also consider the evidence, if it exists, of how these 'non-cognitive' abilities relate to clinical competence too.

The use of such selection assessments, which often have components evaluating both semantic knowledge, and problem-solving skills, are increasingly being used globally. Consequently, our findings have implications for medical selection internationally. That scores from both BMAT and UCAT can predict such distal outcomes adds to the international evidence supporting the use of admissions testing. Furthermore, those sections which assess problem solving or verbal reasoning added most value to the selection process for clinical assessment performance. As many admissions tests in use around the world assess such abilities, such as, for example, the Medical College Admissions Test used in North America, our findings may have implications for medical selection internationally. That being said, research should be undertaken to determine if this indeed is the case in each context locally.

For peer review only

For peer review only - http://bmjopen.bmj.com/site/about/guidelines.xhtml

Closer to home, in the UK, undergraduate medical admissions teams are generally faced with a choice of either BMAT or UCAT as the selection assessment to employ. The present findings do not highlight any clear advantage, in terms of predictive validity, of one over the other. Therefore, choices should be informed by other test qualities, such as the potential impact on widening access and diversifying medicine. In this sense, both BMAT and UCAT have components where performance is associated with certain candidate background factors [7 43]. Thus, at present, in this respect, there would seem to be no overall clear advantage of one selection assessment over the other.

Beyond selection, highlighting those aspects of cognitive intelligence which predict postgraduate clinical simulation performance raises the possibility of targeted interventions for those who score less well on the relevant sections of BMAT or UCAT. However, further research would be required, both in the identification of students for intervention (for example, there may be sociodemographic factors to consider, which have not been accounted in this selection-focussed study), and in the timing and nature of the intervention itself.

Directions for future research

In addition to replicating these findings in other postgraduate exams and subsequent cohorts of medical graduates, there are future opportunities, provided by UKMED, to evaluate the validity of medical selection assessments. In particular, an SJT was added to the UCAT in 2013. Previous research has shown that scores on the SJT do possess some predictive validity for undergraduate supervisor ratings [44]. In this context, it will be important to consider to what extent the SJT scores predict performance in clinical assessments, and other construct-relevant outcomes, and whether this is incremental to other selection measures.

Another change made to the structure of the UCAT has been the replacement of the decision analysis subtest for 'decision making' in 2017. Once the data availability allows, it would be

For peer review only - http://bmjopen.bmj.com/site/about/guidelines.xhtml

important to estimate the relationship between *decision making* and the performance on PACES and other postgraduate practical examinations.

Given that the two selection assessments considered here had comparable predictive validity, as mentioned above, other properties should be considered when choosing such a test for use in medical admissions processes.. It has been highlighted that the trade-off between selecting for future performance and 'adverse impact' on certain underrepresented groups can be conceptualised, and modelled, as a problem of 'pareto-optimisation' [45]. That is, there are optimal, and suboptimal, combinations of how elements within a selection process can be weighted and combined, with the goal of selecting the best doctors while maintaining or improving the diversity of the medical workforce. Such information is vital if selectors are to make informed choices.

Ultimately, linking selection test scores to relevant patient outcomes in clinical practice would be the most desirable source of validity evidence. This would shed light on how, if at all, such assessment scores could predict typical performance, in contrast to the maximal performance observed in high stakes postgraduate clinical exams. Nevertheless, as emphasised early, this is extremely challenging, though more advanced statistical techniques may eventually be able to disentangle environment from individual physician effects. Such approaches would depend on clinicians working across different (ideally small) teams in the hope that the individual doctor's clinical 'footprint' might be observable.

Conclusions

Our findings suggest that both BMAT and UCAT contain components that show some incremental predictive validity for MRCP performance, with broadly similar effect sizes. The subtests *aptitude and skills* and *verbal reasoning* of most value, over and above conventional metrics of educational performance, in this regard. Thus, it could be argued it is these aspects

For peer review only - http://bmjopen.bmj.com/site/about/guidelines.xhtml

of cognitive performance, rather than factual recall, that may be of most value in the selection of future doctors in this context. Both test developers and selectors should consider this evidence when considering how such assessments should be constructed and used within the medical selection process.

LIST OF ABBREVIATIONS

- BMAT: BioMedical Admissions Test
- GCSE: General Certificate of Secondary Education
- MRCP: Membership of the Royal Colleges of Physicians of the UK
- MRCS: Membership of the Royal College of Surgeons
- PACES: Practical Assessment of Clinical Examination Skills
- SJT: Situational Judgement Test
- UCAT: Universities Clinical Aptitude Test
- UKMED: UK Medical Education Database
- USMLE: US Medical Licensing Examination

DECLARATIONS

Ethics approval and consent to participate

Ethical approval for the study was not required as it relied on the analysis of de-identified routinely collected data. This was confirmed in writing by the chair of the University of York's Health Sciences Ethics committee.

Data sharing statement

The dataset supporting the conclusions of this article is available from UKMED on application (www.ukmed.ac.uk).

For peer review only

UK Medical Education Database ("UKMED") P051 extract generated on 13/05/2019. Approved for publication on 20/11/2019. We are grateful to UKMED for the use of these data. However, UKMED bears no responsibility for their analysis or interpretation. The data includes information derived from that collected by the Higher Education Statistics Agency Limited ("HESA") and provided to the GMC ("HESA Data"). Source: HESA Student Record 2002/03 to 2017/18 Copyright Higher Education Statistics Agency Limited. The Higher Education Statistics Agency Limited makes no warranty as to the accuracy of the HESA Data, cannot accept responsibility for any inferences or conclusions derived by third parties from data or other information supplied by it.

Competing interests

UCAT pay for a portion of LWP's research time. LWP and PAT have received travel expenses for attendance at UCAT consortium meetings. PAT has previously received research funding from the UCAT consortium. KYFC is an employee of Cambridge Assessment - a group of exam boards that owns and administers the BioMedical Admissions Test (BMAT); UK GCSEs and A-levels; and International GCSEs and A-levels. ICM is member of various MRCP(UK) committees overseeing and analysing results from MRCP(UK) examinations.

Funding

LWP is partly funded by the UCAT Consortium. The UCAT consortium partly funded this research but did not play an active role in determining the study design or reporting the results.

University College London receives money from MRCP(UK) for the contribution of ICM to the running of the examination, but ICM receives none of that money.

For peer review only - http://bmjopen.bmj.com/site/about/guidelines.xhtml

DS is employed by the GMC as a data analyst working on the UKMED project. The views expressed here are his views and not the views of the GMC.

PAT's research time for this project was funded by an NIHR Career Development Fellowship (CDF-2015-08-11). This paper presents independent research funded by the National Institute for Health Research (NIHR). The views expressed are those of the authors and not necessarily those of the NHS, the NIHR or the Department of Health and Social Care.

Authors contributions

PAT led on conception of the project, with support from LWP, ICM, KYFC and DS. LWP led on data analysis with support from PAT. All authors contributed to interpretation of the results. LWP led on writing of the manuscript, with support from ICM, KYFC, DS and PAT. All authors have approved the final version of the article submitted.

Acknowledgements

We would like to thank Rachel Greatrix at UCAT for her comments on an earlier version of this manuscript.

For peer review only

For peer review only - http://bmjopen.bmj.com/site/about/guidelines.xhtml

TABLES

**Test**	**Subtests**
**BioMedical Admissions Test** [8 10]	**Aptitude and Skills** – 'problem solving and understanding argument'. Replaced by **thinking skills** in 2020.
	**Scientific Knowledge and Applications -** 'the ability to apply scientific knowledge typically covered in school science and mathematics by the age of 16'.
	**Writing - '**the ability to select, develop and organise ideas and to communicate them in writing, concisely and effectively'
**University Clinical Aptitude Test** [9 11]	**Abstract Reasoning -** 'assesses the use of convergent and divergent thinking to infer relationships from information'
	**Decision Making –'**assesses the ability to make sound decisions and judgements using complex information'. Prior to 2017, **decision analysis** was assessed rather than decision making.
	**Quantitative Reasoning -** 'assesses the ability to critically evaluate information presented in numerical form'
	**Verbal Reasoning -** 'assesses the ability to critically evaluate information presented in written form'
	**Situational Judgment Test -** 'measures the capacity to understand real world situations and to identify critical factors and appropriate behaviour in dealing with them'. Introduced in 2013, not considered in this study.
**Membership of the**	**Part 1 -** assess 'a broad range of appropriate knowledge'

For peer review only - http://bmjopen.bmj.com/site/about/guidelines.xhtml

**Royal College of Physicians of the UK** [18 19]	**Part 2 -** test the acquisition of 'a representative sample of medical knowledge, skills and behaviour'
	**Practical Assessment of Clinical Examination Skills -** designed to test 'clinical knowledge and skills', and involves candidates examining real patients with real clinical signs in various organ systems, as well as taking histories and carrying out clinical communication, usually with surrogate patients

Table 1: Description of the three tests considered in this study, alongside a description of the subtests and what they aim to assess.

For peer review only

	All graduates (n=69,885 in total, incl. 20,175 individuals who sat BMAT and 43,685 individuals who sat UCAT)	Entrants who sat both BMAT and UCAT (n=13,505)	Individuals who sat BMAT, UCAT and had data available for at least one section of the MRCP (n=3,045)	Individuals who sat BMAT, UCAT, MRCP, had complete prior educational data and sat A-levels prior to 2010 (n=1,320)
**Age at BMAT**	17.73 (1.67)	17.68 (1.47)	17.62 (1.31)	17.46 (0.92)
**Age at UCAT**	18.59 (2.91)	17.74 (1.54)	17.70 (1.39)	17.47 (0.94)
**Sex**	43.02% male	47.04% male	48.05% male	48.33% male
**BMAT: AaS**	5.18 (1.06)	5.19 (1.13)	5.28 (1.15)	5.28 (1.10)
**BMAT: SKaA**	5.09 (1.00)	5.13 (1.05)	5.24 (1.08)	5.25 (1.04)
**BMAT: W**	8.92 (2.32)	9.20 (2.30)	8.51 (1.98)	8.49 (1.89)
**UCAT: AR**	636.76 (82.36)	648.91 (81.13)	644.42 (80.21)	647.15 (79.41)
**UCAT: DA**	659.44 (89.34)	673.06 (86.54)	666.82 (89.17)	673.58 (88.52)
**UCAT: QR**	668.82 (81.49)	684.76 (80.86)	677.42 (77.87)	687.53 (70.17)
**UCAT: VR**	617.81 (76.48)	626.37 (76.05)	635.07 (78.04)	637.96 (73.33)
**A-level performance, standardised by applicants**	0.04 (0.98)	0.25 (0.79)	0.27 (0.71)	0.20 (0.73)
**GCSE performance, standardised by applicants**	0.29 (0.83)	0.47 (0.80)	0.55 (0.76)	0.65 (0.58)

Table 2. Descriptive statistics across various sub-cohorts of the dataset. Means and standard deviations are presented, except for *sex* where percentages are displayed. AaS = aptitude and skills; SKaA = scientific knowledge and applications; W = writing; AR = abstract reasoning; DA = decision analysis; QR = quantitative reasoning; VR = verbal reasoning.

For peer review only - http://bmjopen.bmj.com/site/about/guidelines.xhtml

REFERENCES

1. McManus I, Woolf K, Dacre J, et al. The Academic Backbone: Longitudinal Continuities in Educational Achievement from Secondary School and Medical School to MRCP(UK) and the Specialist Register in UK Medical Students and Doctors. *BMC Medicine* 2013;11:242.
2. Mercer A, Puddey IB. Admission selection criteria as predictors of outcomes in an undergraduate medical course: a prospective study. *Medical Teacher* 2011;33(12):997-1004.
3. Kim T, Chang J-Y, Myung SJ, et al. Predictors of undergraduate and postgraduate clinical performance: a longitudinal cohort study. *Journal of Surgical Education* 2016;73(4):715-20.
4. Al Alwan I, Al Kushi M, Tamim H, et al. Health sciences and medical college preadmission criteria and prediction of in-course academic performance: a longitudinal cohort study. *Advances in Health Sciences Education* 2013;18(3):427-38.
5. Shulruf B, Poole P, Wang GY, et al. How well do selection tools predict performance later in a medical programme? *Advances in Health Sciences Education* 2012;17(5):615-26.
6. Gupta N, Nagpal G, Dhaliwal U. Student performance during the medical course: role of pre-admission eligibility and selection criteria. *National Medical Journal of India* 2013;26(4):223-6.
7. Tiffin PA, McLachlan JC, Webster LAD, et al. Comparison of the sensitivity of the UKCAT and A Levels to sociodemographic characteristics: a national study. *BMC Medical Education* 2014;14:7.
8. Cambridge Assessment Admissions Testing. The BioMedical Admissions Test (BMAT) [Available from: http://www.admissionstestingservice.org/for-test-takers/bmat/ accessed 26th November 2021.
9. UCAT. About UCAT [Available from: https://www.ucat.ac.uk/about-ucat/ accessed 26th November 2021.
10. Cambridge Assessment Admissions Testing. BMAT - November test format [Available from: https://www.admissionstesting.org/for-test-takers/bmat/bmat-november/test-format/ accessed 26th November 2021.
11. UCAT. UCAT Test Format [Available from: https://www.ucat.ac.uk/ucat/test-format/. Accessed 26th November 2021.

For peer review only - http://bmjopen.bmj.com/site/about/guidelines.xhtml

12. Emery JL, Bell JF. The predictive validity of the BioMedical Admissions Test for pre-clinical examination performance. *Medical Education* 2009;43(6):557-64.
13. Tiffin PA, Mwandigha LM, Paton LW, et al. Predictive validity of the UKCAT for medical school undergraduate performance: a national prospective cohort study. *BMC Medicine* 2016;14(1):140.
14. McManus I, Dewberry C, Nicholson S, et al. The UKCAT-12 Study: Educational Attainment, Aptitude Test Performance, Demographic and Socio-Economic Contextual Factors as Predictors of First Year Outcome in a Collaborative Study of Twelve UK Medical Schools. *BMC Medicine* 2013;11:244.
15. MacKenzie R, Cleland J, Ayansina D, et al. Does the UKCAT predict performance on exit from medical school? A national cohort study. *BMJ Open* 2016;6(10):e011313.
16. McManus IC, Woolf K, Harrison D, et al. Calculated grades, predicted grades, forecasted grades and actual A-level grades: Reliability, correlations and predictive validity in medical school applicants, undergraduates, and postgraduates in a time of COVID-19. *medRxiv* 2020
17. Ellis R, Brennan P, Scrimgeour DS, et al. Performance at medical school selection correlates with success in Part A of the intercollegiate Membership of the Royal College of Surgeons (MRCS) examination. *Postgraduate Medical Journal* 2021
18. Royal Colleges of Physicians of the United Kingdom. MRCPUK [Available from: https://www.mrcpuk.org/. Accessed 26th November 2021.
19. Royal College of Physicians of the United Kingdom. MRCP(UK) examinations [Available from: https://www.mrcpuk.org/mrcpuk-examinations accessed 26th November 2021.
20. Dowell J, Cleland J, Fitzpatrick S, et al. The UK medical education database (UKMED) what is it? Why and how might you use it? *BMC Medical Education* 2018;18(1):6.
21. Flanagan DP, Alfonso VC, Ortiz SO, et al. Cognitive assessment: Progress in psychometric theories of intelligence, the structure of cognitive ability tests, and interpretive approaches to cognitive test performance. In Saklofske, DP, Reynolds, CR & Schwean, VL (Eds.) *The Oxford Handbook of Child Psychological Assessment* (pp. 239-285). 2013
22. UK Medical Education Database. Process for completing UKMED research - version 5 2020 [Available from: https://www.ukmed.ac.uk/documents/UKMED_research_process.pdf. Accessed 26th November 2021.

For peer review only - http://bmjopen.bmj.com/site/about/guidelines.xhtml

23. McManus I, Ludka K. Resitting a high-stakes postgraduate medical examination on multiple occasions: nonlinear multilevel modelling of performance in the MRCP(UK) examinations. *BMC Medicine* 2012;10:60.

24. Tiffin PA. Understanding the Dimensionality and Reliability of the Cognitive Scales of the UK Clinical Aptitude test (UKCAT): Summary Version of the Report 2013 [Available from: https://www.ucat.ac.uk/media/1182/understanding-the-dimensionality-and-reliability-of-the-cognitive-scales-of-the-ukcat.pdf Accessed 26th November 2021.

25. McManus I, Elder A, De Champlain A, et al. Graduates of different UK medical schools show substantial differences in performance on MRCP(UK) Part 1, Part 2 and PACES examinations. *BMC Medicine* 2008;6:5.

26. Stata 14 for Windows [program]. College Station, TX: StataCorp.

27. Cheung KYF, McElwee S. What skills are we assessing? Cognitive validity in BMAT. In: Cheung KYF, McElwee S, Emery J, eds. Applying the socio-cognitive framework to the BioMedical Admissions Test (BMAT): Insights from language assessment. Cambridge, UK: Cambridge University Press 2017:35-80.

28. Cambridge Assessment International Education. Cambridge International AS and A Level Thinking Skills (9694) [Available from: https://www.cambridgeinternational.org/programmes-and-qualifications/cambridge-international-as-and-a-level-thinking-skills-9694/ accessed 26th November 2021.

29. Mwandigha LM, Tiffin PA, Paton LW, et al. What is the effect of secondary (high) schooling on subsequent medical school performance? A national, UK-based, cohort study. *BMJ Open* 2018;8(5)

30. Tiffin PA, Finn GM, McLachlan JC. Evaluating professionalism in medical undergraduates using selected response questions: findings from an item response modelling study. *BMC Medical Education* 2011;11:43.

31. Conrad N, Patry M. Conscientiousness and Academic Performance: A Mediational Analysis. *International Journal for the Scholarship of Teaching and Learning* 2012;6

32. Demange PA, Malanchini M, Mallard TT, et al. Investigating the genetic architecture of noncognitive skills using GWAS-by-subtraction. *Nature Genetics* 2021;53(1):35-44.

33. Patterson F, Tiffin PA, Lopes S, et al. Unpacking the dark variance of differential attainment on examinations in overseas graduates. *Medical Education* 2018;52(7):736-46.

For peer review only - http://bmjopen.bmj.com/site/about/guidelines.xhtml

34. Joseph D, Newman D. Emotional Intelligence: An Integrative Meta-Analysis and Cascading Model. *Journal of Applied Psychology* 2010;95:54-78.

35. MacCann C, Joseph DL, Newman DA, et al. Emotional intelligence is a second-stratum factor of intelligence: Evidence from hierarchical and bifactor models. *Emotion* 2014;14(2):358-74.

36. Matthews G, Zeidner M, Roberts R. Appendix A: A Review and Critique of Social Intelligence. Emotiional Intelligence: Science and Myth. Cambridge, MA: The MIT Press 2000:551-61.

37. Tiffin PA, Paton LW, Mwandigha LM, et al. Predicting fitness to practise events in international medical graduates who registered as UK doctors via the Professional and Linguistic Assessments Board (PLAB) system: a national cohort study. *BMC Medicine* 2017;15(1):66.

38. Kane MT. Validating the Interpretations and Uses of Test Scores. *Journal of Educational Measurement* 2013;50(1):1-73.

39. UCAT. University Clinical Aptitude Test [Available from: https://www.ucat.ac.uk/ accessed 26th November 2021.

40. Elder AT, McManus IC, Patrick A, et al. The value of the physical examination in clinical practice: an international survey. *Clinical Medicine* 2017;17(6):490.

41. Hausknecht JP, Halpert JA, Di Paolo NT, et al. Retesting in selection: a meta-analysis of coaching and practice effects for tests of cognitive ability. *Journal of Applied Psychology* 2007;92(2):373.

42. Webster ES, Paton LW, Crampton PES, et al. Situational judgement test validity for selection: A systematic review and meta-analysis. *Medical Education* 2020;54(10):888-902.

43. Emery JL, Bell JF, Vidal Rodeiro CL. The BioMedical admissions test for medical student selection: issues of fairness and bias. *Medical Teacher* 2011;33(1):62-71.

44. Patterson F, Cousans F, Edwards H, et al. The Predictive Validity of a Text-Based Situational Judgment Test in Undergraduate Medical and Dental School Admissions. *Academic Medicine* 2017

45. De Corte W, Sackett P, Lievens F. Designing Pareto-Optimal Selection Systems: Formalizing the Decisions Required for Selection System Development. *Journal of Applied Psychology* 2011;96:907-26.

For peer review only - http://bmjopen.bmj.com/site/about/guidelines.xhtml

FIGURE LEGENDS

Figure 1. The flow of data through this study.

Figure 2. Predictive validity of each standardised section score of BMAT for passing each section of the MRCP at the first attempt. Results are shown both unadjusted and adjusted for prior educational performance. AaS = aptitude and skills; SKaA = scientific knowledge and applications; W = writing task.

Figure 3. Predictive validity of each standardised section score of the UCAT for passing each section of the MRCP at the first attempt. Results are shown both unadjusted and adjusted for prior educational performance. AR = abstract reasoning; DA = decision analysis; QR = quantitative reasoning; VR = verbal reasoning.

For peer review only

For peer review only - http://bmjopen.bmj.com/site/about/guidelines.xhtml

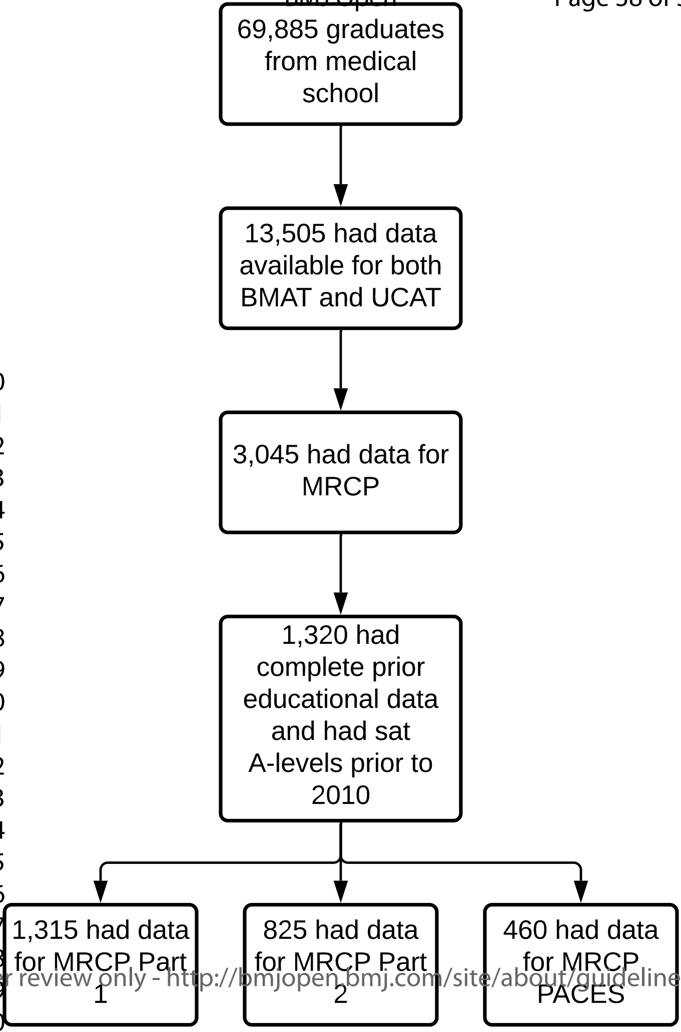
69,885 graduates from medical school
13,505 had data available for both BMAT and UCAT
3,045 had data for MRCP
1,320 had complete prior educational data and had sat A-levels prior to 2010
1,315 had data for MRCP Part 1
825 had data for MRCP Part 2
460 had data for MRCP PACES

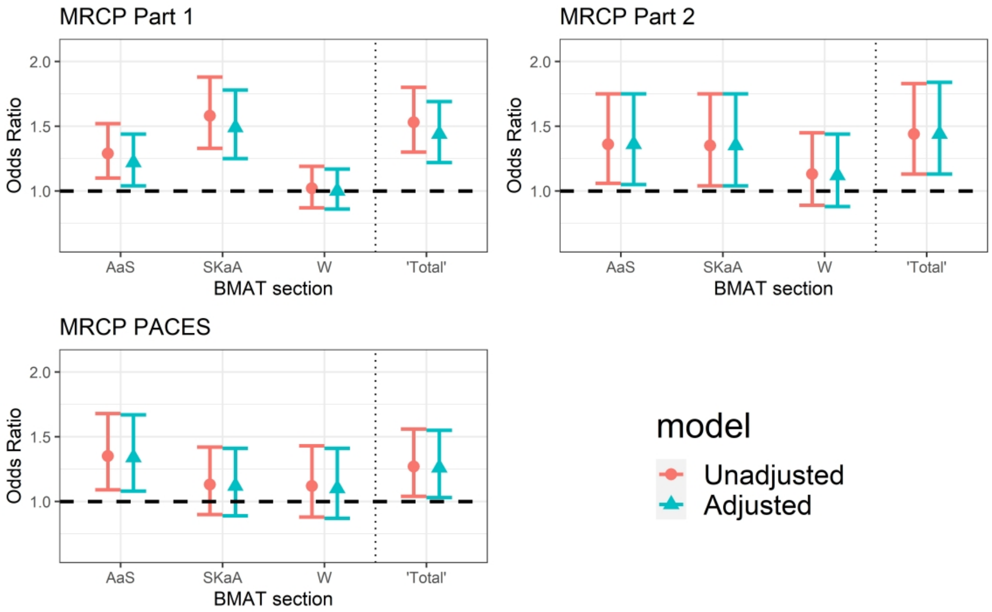

Figure 2. Predictive validity of each standardised section score of BMAT for passing each section of the MRCP at the first attempt. Results are shown both unadjusted and adjusted for prior educational performance. AaS = aptitude and skills; SKaA = scientific knowledge and applications; W = writing task.

202x125mm (600 x 600 DPI)

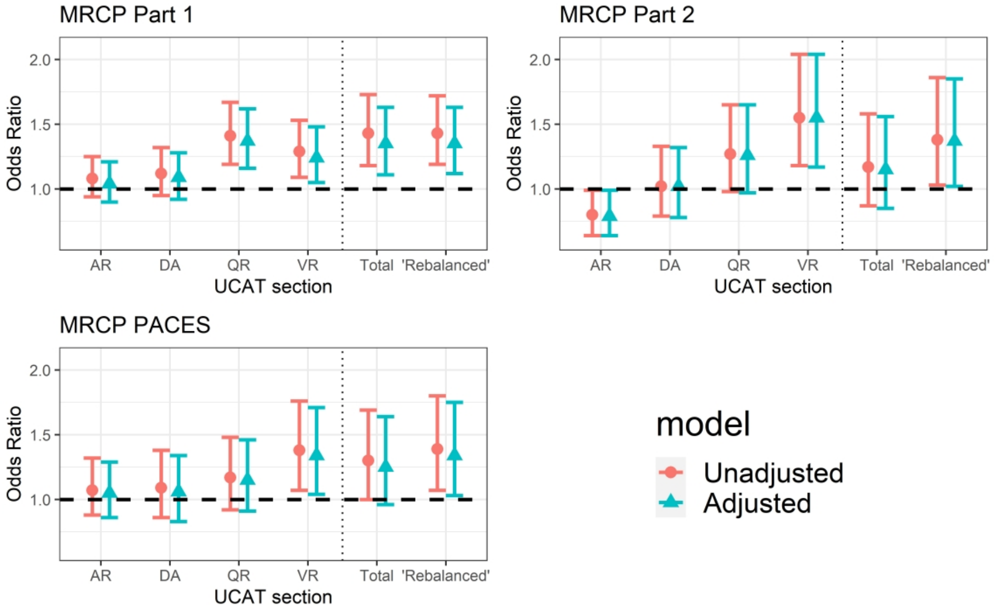

Figure 3. Predictive validity of each standardised section score of the UCAT for passing each section of the MRCP at the first attempt. Results are shown both unadjusted and adjusted for prior educational performance. AR = abstract reasoning; DA = decision analysis; QR = quantitative reasoning; VR = verbal reasoning.

202x125mm (600 x 600 DPI)

Supplemental Material for 'Can achievement at medical admissions tests predict future performance in postgraduate clinical assessments? A national cohort study'

Lewis W Paton, I C McManus, Kevin Y F Cheung, Daniel S Smith, Paul A Tiffin

A: Passing each section of the MRCP at the first attempt

Tables A1 – A3 display the results for models predicting passing each section of the Membership of the Royal Colleges of Physicians of the UK (MRCP) at the first attempt. These results are also depicted in Figures 2 and 3.

Pass at 1st attempt	MRCP: Part 1	
	Unadjusted ORs	ORs adjusted for PEA
**BMAT**		
**Aptitude and Skills**	1.29 (1.10 to 1.52) p=0.002	1.22 (1.04 to 1.44) p=0.02
**Scientific Knowledge and Applications**	1.58 (1.33 to 1.88) p<0.001	1.49 (1.25 to 1.78) p<0.001
**Writing**	1.02 (0.87 to 1.19) p=0.84	1.00 (0.86 to 1.17) p=0.98
**'Total'**	1.53 (1.30 to 1.80) p<0.001	1.44 (1.22 to 1.69) p<0.001
**UCAT**		
**Abstract Reasoning**	1.08 (0.94 to 1.25) p=0.28	1.04 (0.90 to 1.21) p=0.56
**Decision Analysis**	1.12 (0.95 to 1.32) p=0.17	1.09 (0.92 to 1.28) p=0.32
**Quantitative Reasoning**	1.41 (1.19 to 1.67) p<0.001	1.37 (1.16 to 1.62) p<0.001
**Verbal Reasoning**	1.29 (1.09 to 1.53) p=0.003	1.24 (1.05 to 1.48) p=0.01
**Total**	1.43 (1.18 to 1.73) p<0.001	1.35 (1.11 to 1.63) p=0.003
**'Rebalanced' total**	1.43 (1.19 to 1.72) p<0.001	1.35 (1.12 to 1.63) p<0.01

Table A1. Results from multilevel logistic regression models predicting passing *MRCP: Part 1* at the first attempt, unadjusted and adjusted for prior educational attainment (PEA).

For peer review only

Pass at 1st attempt	MRCP: Part 2	
	**Unadjusted ORs**	**ORs adjusted for PEA**
**BMAT**		
**Aptitude and Skills**	1.36 (1.06 to 1.75) p=0.02	1.36 (1.05 to 1.75) p=0.02
**Scientific Knowledge and Applications**	1.35 (1.04 to 1.75) p=0.02	1.35 (1.04 to 1.75) p=0.02
**Writing**	1.13 (0.89 to 1.45) p=0.32	1.12 (0.88 to 1.44) p=0.36
**'Total'**	1.44 (1.13 to 1.83) p<0.01	1.44 (1.13 to 1.84) p<0.01
**UCAT**		
**Abstract Reasoning**	0.80 (0.64 to 0.99) p=0.04	0.79 (0.64 to 0.99) p=0.04
**Decision Analysis**	1.02 (0.79 to 1.33) p=0.86	1.02 (0.78 to 1.32) p=0.91
**Quantitative Reasoning**	1.27 (0.98 to 1.65) p=0.07	1.26 (0.97 to 1.65) p=0.08
**Verbal Reasoning**	1.55 (1.18 to 2.04) p=0.002	1.55 (1.17 to 2.04) p<0.01
**Total**	1.17 (0.87 to 1.58) p=0.30	1.15 (0.85 to 1.56) p=0.36
**'Balanced' total**	1.38 (1.03 to 1.86) p=0.03	1.37 (1.02 to 1.85) p=0.04

Table A2. Results from multilevel logistic regression models predicting passing *MRCP: Part 2* at the first attempt, unadjusted and adjusted for prior educational attainment (PEA).

For peer review only

Pass at 1st attempt	MRCP: PACES	
	Unadjusted ORs	ORs adjusted for PEA
**BMAT**		
**Aptitude and Skills**	1.35 (1.09 to 1.68) p=0.01	1.34 (1.08 to 1.67) p=0.01
**Scientific Knowledge and Applications**	1.13 (0.90 to 1.42) p=0.30	1.12 (0.89 to 1.41) p=0.34
**Writing**	1.12 (0.88 to 1.43) p=0.34	1.10 (0.87 to 1.41) p=0.42
**'Total'**	1.27 (1.04 to 1.56) p=0.02	1.26 (1.03 to 1.55) p=0.03
**UCAT**		
**Abstract Reasoning**	1.07 (0.88 to 1.32) p=0.49	1.05 (0.86 to 1.29) p=0.63
**Decision Analysis**	1.09 (0.86 to 1.38) p=0.47	1.06 (0.83 to 1.34) p=0.66
**Quantitative Reasoning**	1.17 (0.92 to 1.48) p=0.20	1.15 (0.91 to 1.46) p=0.25
**Verbal Reasoning**	1.38 (1.07 to 1.76) p=0.01	1.34 (1.04 to 1.71) p=0.02
**Total**	1.30 (>1.00 to 1.69) p=0.05	1.25 (0.96 to 1.64) p=0.10
**'Rebalanced' total**	1.39 (1.07 to 1.80) p=0.01	1.34 (1.03 to 1.75) p=0.03

Table A3. Results from multilevel logistic regression models predicting passing *MRCP: PACES* at the first attempt, unadjusted and adjusted for prior educational attainment (PEA).

For peer review only

B: Imputed results

Tables B1-B3 display results from multilevel logistic models fitted to both non-imputed and imputed data. These models are adjusted for prior educational attainment.

**Pass at 1st attempt**	**MRCP: Part 1**	
	**Adjusted ORs**	
	**Non-imputed**	**Imputed**
	**BMAT**	
**Aptitude and skills**	1.22 (1.04 to 1.44)<br>p=0.02	1.25 (1.08 to 1.45)<br>p<0.01
**Scientific knowledge and applications**	1.49 (1.25 to 1.78)<br>p<0.001	1.52 (1.30 to 1.78)<br>p<0.001
**Writing**	1.00 (0.86 to 1.17)<br>p=0.98	1.08 (0.94 to 1.24)<br>p=0.30
**'Total'**	1.44 (1.22 to 1.69)<br>p<0.001	1.48 (1.28 to 1.71)<br>p<0.001
	**UCAT**	
**Abstract Reasoning**	1.04 (0.90 to 1.21)<br>p=0.56	1.07 (0.98 to 1.17)<br>p=0.14
**Decision Analysis**	1.09 (0.92 to 1.28)<br>p=0.32	1.10 (<1.00 to 1.21)<br>p=0.06
**Quantitative Reasoning**	1.37 (1.16 to 1.62)<br>p<0.001	1.31 (1.18 to 1.45)<br>p<0.001
**Verbal Reasoning**	1.24 (1.05 to 1.48)<br>p=0.01	1.30 (1.17 to 1.43)<br>p<0.001
**Total**	1.35 (1.11 to 1.63)<br>p=0.003	1.37 (1.22 to 1.54)<br>p<0.001
**'Rebalanced' total**	1.35 (1.12 to 1.63)<br>p<0.01	1.40 (1.25 to 1.56)<br>p<0.001

Table B1. Comparison of odds ratios on non-imputed and imputed data, predicting passing *MRCP: Part 1* at the first attempt. All models are adjusted for prior educational attainment.

For peer review only

Pass at 1st attempt	MRCP: Part 2	
	Adjusted ORs	
	Non-imputed	Imputed
**BMAT**		
**Aptitude and skills**	1.36 (1.05 to 1.75) p=0.02	1.35 (1.09 to 1.67) p=0.01
**Scientific knowledge and applications**	1.35 (1.04 to 1.75) p=0.02	1.33 (1.06 to 1.65) p=0.01
**Writing**	1.12 (0.88 to 1.44) p=0.36	1.14 (0.92 to 1.40) p=0.24
**'Total'**	1.44 (1.13 to 1.84) p<0.01	1.40 (1.13 to 1.72) p<0.01
**UCAT**		
**Abstract Reasoning**	0.79 (0.64 to 0.99) p=0.04	1.00 (0.87 to 1.16) p=0.97
**Decision Analysis**	1.02 (0.78 to 1.32) p=0.91	0.98 (0.83 to 1.12) p=0.66
**Quantitative Reasoning**	1.26 (0.97 to 1.65) p=0.08	1.22 (1.04 to 1.44) p=0.02
**Verbal Reasoning**	1.55 (1.17 to 2.04) p=0.002	1.47 (1.25 to 1.74) p<0.001
**Total**	1.15 (0.85 to 1.56) p=0.36	1.26 (1.05 to 1.52) p=0.01
**'Rebalanced' total**	1.37 (1.02 to 1.85) p=0.04	1.42 (1.19 to 1.70) p<0.001

Table B2. Comparison of odds ratios on non-imputed and imputed data, predicting passing *MRCP: Part 2* at the first attempt. All models are adjusted for prior educational attainment.

For peer review only

Pass at 1st attempt	MRCP: PACES	
	Adjusted ORs	
	Non-imputed	Imputed
**BMAT**		
**Aptitude and skills**	1.34 (1.08 to 1.67) p=0.01	1.20 (1.03 to 1.40) p=0.02
**Scientific knowledge and applications**	1.12 (0.89 to 1.41) p=0.34	1.12 (0.94 to 1.35) p=0.21
**Writing**	1.10 (0.87 to 1.41) p=0.42	1.00 (0.84 to 1.19) p=0.99
**'Total'**	1.26 (1.03 to 1.55) p=0.03	1.19 (1.03 to 1.37) p=0.02
**UCAT**		
**Abstract Reasoning**	1.05 (0.86 to 1.29) p=0.63	1.10 (0.97 to 1.24) p=0.13
**Decision Analysis**	1.06 (0.83 to 1.34) p=0.66	1.14 (>1.00 to 1.30) p=0.05
**Quantitative Reasoning**	1.15 (0.91 to 1.46) p=0.25	1.07 (0.94 to 1.23) p=0.31
**Verbal Reasoning**	1.34 (1.04 to 1.71) p=0.02	1.15 (>1.00 to 1.32) p=0.05
**Total**	1.25 (0.96 to 1.64) p=0.10	1.22 (1.05 to 1.42) p=0.01
**'Rebalanced' total**	1.34 (1.03 to 1.75) p=0.03	1.22 (1.05 to 1.41) p=0.01

Table B3. Comparison of odds ratios on non-imputed and imputed data, predicting passing *MRCP: PACES* at the first attempt. All models are adjusted for prior educational attainment.

For peer review only

C: Results from 'score relative to pass'

Tables C1 – C3 display results from linear regression models predicting score relative to pass at each section of the MRCP(UK) at the first attempt. Regression coefficients are shown unadjusted and adjusted for prior educational attainment.

Score relative to pass	MRCP: Part 1	
	Unadjusted β	β adjusted for PEA
**BMAT**		
**Aptitude and Skills**	1.25 (0.68 to 1.82) p<0.001	1.07 (0.49 to 1.65) p<0.001
**Scientific Knowledge and Applications**	2.02 (1.43 to 2.60) p<0.001	1.83 (1.24 to 2.42) p<0.001
**Writing**	-0.04 (-0.60 to 0.51) p=0.88	-0.06 (-0.62 to 0.49) p=0.83
**'Total'**	1.95 (1.40 to 2.50) p<0.001	1.76 (1.20 to 2.32) p<0.001
**UCAT**		
**Abstract Reasoning**	0.25 (-0.25 to 0.75) p=0.38	0.14 (-0.36 to 0.64) p=0.59
**Decision Analysis**	0.46 (-0.10 to 1.03) p=0.11	0.37 (-0.19 to 0.94) p=0.19
**Quantitative Reasoning**	1.62 (1.03 to 2.21) p<0.001	1.51 (0.92 to 2.10) p<0.001
**Verbal Reasoning**	1.03 (0.43 to 1.62) p<0.001	0.91 (0.31 to 1.50) p<0.01
**Total**	1.46 (0.80 to 2.11) p<0.001	1.27 (0.61 to 1.93) p<0.001
**'Rebalanced' total**	1.47 (0.82 to 2.12) p<0.001	1.30 (0.64 to 1.95) p<0.001

Table C1. Results from multilevel linear regression models predicting score relative to pass on *MRCP: Part 1* at the first attempt, unadjusted and adjusted for prior educational attainment (PEA).

For peer review only

Score relative to pass	MRCP: Part 2	
	Unadjusted β	β adjusted for PEA
**BMAT**		
**Aptitude and Skills**	1.80 (1.27 to 2.32) p<0.001	1.75 (1.21 to 2.28) p<0.001
**Scientific Knowledge and Applications**	1.71 (1.16 to 2.26) p<0.001	1.71 (1.16 to 2.27) p<0.001
**Writing**	0.30 (-0.26 to 0.87) p=0.29	0.38 (-0.19 to 0.95) p=0.19
**'Total'**	1.90 (1.42 to 2.38) p<0.001	1.87 (1.37 to 2.36) p<0.001
**UCAT**		
**Abstract Reasoning**	0.05 (-0.45 to 0.55) p=0.84	0.04 (-0.47 to 0.55) p=0.87
**Decision Analysis**	0.37 (-0.21 to 0.95) p=0.21	0.38 (-0.20 to 0.97) p=0.20
**Quantitative Reasoning**	1.14 (0.53 to 1.74) p<0.001	1.17 (0.56 to 1.78) p<0.001
**Verbal Reasoning**	1.66 (1.04 to 2.27) p<0.001	1.62 (1.00 to 2.25) p<0.001
**Total**	1.22 (0.56 to 1.88) p<0.001	1.25 (0.58 to 1.92) p<0.001
**'Rebalanced' total**	1.60 (0.95 to 2.26) p<0.001	1.69 (1.03 to 2.36) p<0.001

Table C2. Results from multilevel linear regression models predicting score relative to pass on *MRCP: Part 2* at the first attempt, unadjusted and adjusted for prior educational attainment (PEA).

For peer review only

Score relative to pass	MRCP: PACES	
	Unadjusted β	β adjusted for PEA
**BMAT**		
**Aptitude and Skills**	2.37 (0.88 to 2.89) p<0.01	2.36 (0.85 to 3.87) p<0.01
**Scientific Knowledge and Applications**	1.68 (0.11 to 3.36) p=0.04	1.66 (0.07 to 3.26) p=0.04
**Writing**	1.22 (-0.47 to 2.92) p=0.16	1.17 (-0.54 to 2.88) p=0.18
**'Total'**	2.19 (0.80 to 3.57) p<0.01	2.19 (0.78 to 3.60) p<0.01
**UCAT**		
**Abstract Reasoning**	0.68 (-0.77 to 2.14) p=0.36	0.62 (-0.85 to 2.10) p=0.41
**Decision Analysis**	0.86 (-0.84 to 2.55) p=0.32	0.77 (-0.96 to 2.50) p=0.38
**Quantitative Reasoning**	0.79 (-0.91 to 2.49) p=0.36	0.74 (-0.97 to 2.44) p=0.40
**Verbal Reasoning**	3.00 (1.33 to 4.67) p<0.001	2.99 (1.28 to 4.70) p<0.001
**Total**	2.18 (0.36 to 4.00) p=0.02	2.14 (0.26 to 4.02) p=0.03
**'Rebalanced' total**	2.88 (1.09 to 4.66) p<0.01	2.89 (1.04 to 4.74) p<0.01

Table C3. Results from multilevel linear regression models predicting score relative to pass on *MRCP: PACES* at the first attempt, unadjusted and adjusted for prior educational attainment (PEA).

For peer review only

STROBE 2007 (v4) Statement—Checklist of items that should be included in reports of *cohort studies*

Section/Topic	Item #	Recommendation	Reported on page #
**Title and abstract**	1	(*a*) Indicate the study's design with a commonly used term in the title or the abstract	1
		(*b*) Provide in the abstract an informative and balanced summary of what was done and what was found	2
**Introduction**			
Background/rationale	2	Explain the scientific background and rationale for the investigation being reported	4-7
Objectives	3	State specific objectives, including any prespecified hypotheses	7
**Methods**			
Study design	4	Present key elements of study design early in the paper	8, Fig 1
Setting	5	Describe the setting, locations, and relevant dates, including periods of recruitment, exposure, follow-up, and data collection	8, Fig 1
Participants	6	(*a*) Give the eligibility criteria, and the sources and methods of selection of participants. Describe methods of follow-up	8, Fig 1
		(*b*) For matched studies, give matching criteria and number of exposed and unexposed	n/a
Variables	7	Clearly define all outcomes, exposures, predictors, potential confounders, and effect modifiers. Give diagnostic criteria, if applicable	8-10
Data sources/ measurement	8*	For each variable of interest, give sources of data and details of methods of assessment (measurement). Describe comparability of assessment methods if there is more than one group	8-10
Bias	9	Describe any efforts to address potential sources of bias	9-11
Study size	10	Explain how the study size was arrived at	8-11, Fig 1
Quantitative variables	11	Explain how quantitative variables were handled in the analyses. If applicable, describe which groupings were chosen and why	8-10
Statistical methods	12	(*a*) Describe all statistical methods, including those used to control for confounding	10-11
		(*b*) Describe any methods used to examine subgroups and interactions	10-11
		(*c*) Explain how missing data were addressed	11
		(*d*) If applicable, explain how loss to follow-up was addressed	n/a
		(*e*) Describe any sensitivity analyses	11
**Results**			

For peer review only

Participants	13*	(a) Report numbers of individuals at each stage of study—eg numbers potentially eligible, examined for eligibility, confirmed eligible, included in the study, completing follow-up, and analysed	Fig 1
		(b) Give reasons for non-participation at each stage	n/a
		(c) Consider use of a flow diagram	Fig 1
Descriptive data	14*	(a) Give characteristics of study participants (eg demographic, clinical, social) and information on exposures and potential confounders	Table 2
		(b) Indicate number of participants with missing data for each variable of interest	Table 2
		(c) Summarise follow-up time (eg, average and total amount)	n/a
Outcome data	15*	Report numbers of outcome events or summary measures over time	13
Main results	16	(*a*) Give unadjusted estimates and, if applicable, confounder-adjusted estimates and their precision (eg, 95% confidence interval). Make clear which confounders were adjusted for and why they were included	Fig 2, 3, Supplementary appendix
		(*b*) Report category boundaries when continuous variables were categorized	n/a
		(*c*) If relevant, consider translating estimates of relative risk into absolute risk for a meaningful time period	n/a
Other analyses	17	Report other analyses done—eg analyses of subgroups and interactions, and sensitivity analyses	14, supplementary appendix
**Discussion**			
Key results	18	Summarise key results with reference to study objectives	15-16
**Limitations**			
Interpretation	20	Give a cautious overall interpretation of results considering objectives, limitations, multiplicity of analyses, results from similar studies, and other relevant evidence	15-19
Generalisability	21	Discuss the generalisability (external validity) of the study results	16-20
**Other information**			
Funding	22	Give the source of funding and the role of the funders for the present study and, if applicable, for the original study on which the present article is based	27-28

*Give information separately for cases and controls in case-control studies and, if applicable, for exposed and unexposed groups in cohort and cross-sectional studies.

Note: An Explanation and Elaboration article discusses each checklist item and gives methodological background and published examples of transparent reporting. The STROBE checklist is best used in conjunction with this article (freely available on the Web sites of PLoS Medicine at http://www.plosmedicine.org/, Annals of Internal Medicine at http://www.annals.org/, and Epidemiology at http://www.epidem.com/). Information on the STROBE Initiative is available at www.strobe-statement.org.

For peer review only
